# An Interpretable and Edge Deployable Spatio-Temporal Trajectory Prediction for Autonomous Driving

**DOI:** 10.3390/s26154692

**Published:** 2026-07-23

**Authors:** Rajesh Kannan Megalingam, Naveen Prasaad Selvarajan, Pritty Vijay

**Affiliations:** Sustainable Mobility and Automotive Research Technology Centre (SMART), Department of Electronics and Communication Engineering, Amrita Vishwa Vidyapeetham, Amritapuri, Kollam 690525, Kerala, India; naveenps@am.amrita.edu (N.P.S.); am.sc.p2ari24011@am.students.amrita.edu (P.V.)

**Keywords:** spatio-temporal trajectory prediction, Explainable Artificial Intelligence (XAI), edge AI, lane-aware graph reasoning, behavior-aware motion prediction, embedded autonomous systems

## Abstract

Trajectory prediction is a critical component of autonomous driving systems, enabling vehicles to anticipate future motion behaviors and perform safe decision-making in dynamic traffic environments. While recent trajectory forecasting methods achieve state-of-the-art prediction accuracy, many operate as black-box systems and are evaluated primarily on high-end computing platforms, limiting their interpretability and practical deployment feasibility in resource-constrained autonomous driving systems. To address these limitations, this work proposes an interpretable and edge-deployable spatio-temporal trajectory prediction framework for autonomous driving. The proposed architecture integrates a Temporal Convolutional Network with Multi-Head Self-Attention (TCN–MHSA) in ActorNet for selective temporal modeling, a Lane Graph Attention Network (LaneGAT) for structured spatial reasoning, and a multi-stage FusionNet for actor–lane interaction. To improve model interpretability, a comprehensive Explainable AI (XAI) evaluation framework is introduced, including temporal sensitivity analysis, interaction-aware perturbation studies, spatial influence analysis, and gradient-based feature attribution methods. These analyses provide insights into how the model captures temporal motion dependencies, neighboring vehicle interactions, and environmental context during trajectory prediction. To improve the robustness of the interpretability analysis, temporal sensitivity was additionally evaluated over 100 validation scenes, demonstrating that recent observations consistently exert the greatest influence on trajectory prediction, while neighboring interaction effects gradually diminish with increasing spatial separation. Furthermore, practical real-world deployment feasibility is investigated on the NVIDIA Jetson Xavier NX platform using edge-aware optimization strategies, including mixed-precision inference and graph-complexity reduction techniques for efficient resource-constrained inference, achieving 125.74 ms latency at 12.86 W. Additional edge deployment comparisons with HiVT and SIMPL approaches under identical hardware conditions demonstrate that the proposed framework provides a more favorable balance between computational efficiency and embedded deployment performance. Experimental evaluation on the Argoverse 1 dataset demonstrates a minimum Average Displacement Error (minADE) of 0.90 m, a minimum Final Displacement Error (minFDE) of 1.50 m, a Miss Rate (MR) of 0.19, and DAC = 0.95, while establishing an accuracy–deployability operating point under embedded hardware constraints with low power consumption and practical inference throughput.

## 1. Introduction

Trajectory prediction for autonomous vehicles has evolved from conventional model-based methods to advanced deep learning approaches. Early probabilistic methods involved maneuver-based approaches [1], which make use of pre-defined maneuvers such as lane-following and turning, and intention-based approaches [2], which concentrate on motion pattern analysis. Kalman filtering is another common algorithm [3]; however, since they depend upon linear approximations, they prove inefficient in complex cases. With advancements in deep learning, Recurrent Neural Networks have been used for sequence prediction purposes. Social models [4] added social pooling to account for the impact of surrounding agents on an agent’s future trajectory. Generative models such as DESIRE [5] and structure-based Recurrent Neural Network (RNN) [6] improved prediction quality further. Socially Acceptable Trajectories with Social-Generative Adversarial Networks (Social-GAN) [7] and Social and Physical attention-based model (SoPhie) [8] incorporated adversarial learning and attention mechanisms, achieving notable improvements. Most of these approaches rely on rasterization-based scene representations, causing loss of fine-grained lane geometry critical for accurate trajectory forecasting.

In order to overcome these drawbacks, graph-based as well as vectorization techniques have been developed for better representation of structured roads. VectorNet [9] utilized vectorized map encoding for lane structure preservation. On the other hand, Lane Graph Convolutional Network (LaneGCN) [10] captured interaction among agents and lane graph using graph convolution. Hierarchical and dense trajectories were proposed in DenseTNT (Target-driven trajectory) [11] and Hierarchical vector transformer (HiVT) [12] to improve spatial reasoning. Despite all these advancements, many of the current works are still limited to basic temporal encoders that do not focus on temporal events related to behavior transitions. For capturing the long-range dependencies, transformer-based architectures, including AgentFormer [13], Scene Transformer [14], Motion Transformer (MTR) [15], and Wayformer [16], have been explored and have demonstrated impressive performance using attention mechanisms. However, the major drawback of these techniques is their computational complexity and inability to run in constrained real-time scenarios.

Despite significant progress in trajectory forecasting, several important limitations remain insufficiently explored in existing literature. Existing studies based on recurrent architectures such as Long Short-Term Memory (LSTM) networks [17] and multi-task learning approaches [18] have investigated trajectory and behavior prediction; however, many of these methods still treat forecasting and maneuver understanding as loosely coupled tasks without explicitly analyzing the behavioral importance of temporal observations. Consequently, behavior-critical motion transitions such as lane adjustment and maneuver initiation are not sufficiently interpreted during prediction. In addition, most state-of-the-art trajectory prediction methods are developed and evaluated primarily on server-grade GPUs, while practical deployment feasibility on embedded autonomous driving hardware remains largely unexplored, particularly for graph-based forecasting architectures involving dynamic interaction modeling. Furthermore, despite recent advances in Explainable AI for autonomous driving, limited prior work provides comprehensive interpretability analysis encompassing temporal sensitivity, spatial perturbation, neighboring interaction influence, and feature-level attribution within a deployable trajectory forecasting framework.

To address these limitations, this work proposes an interpretable and edge-deployable spatio-temporal trajectory prediction framework for autonomous driving. The proposed approach integrates temporal importance modeling with lane-aware graph interaction reasoning to capture behavior-relevant motion dynamics in structured traffic environments. Furthermore, the framework is evaluated through explainability-driven analyses and validated on embedded edge hardware to investigate practical deployment feasibility under resource-constrained conditions.

Unlike many recent trajectory forecasting studies that primarily optimize displacement error, the objective of the proposed framework is to investigate a practical operating point that jointly considers three complementary design objectives: forecasting accuracy, model interpretability, and embedded deployment feasibility. These objectives are often conflicting, since improving prediction accuracy typically requires larger and computationally more expensive models, whereas embedded deployment and explainability favor compact and transparent architectures. Consequently, the proposed framework intentionally balances these competing objectives rather than optimizing a single evaluation metric. The effectiveness of this design philosophy is subsequently analyzed through comparative forecasting performance, explainability experiments, and embedded deployment evaluation in Section 4 and Section 5.

The major contributions of this work are summarized as follows:

- An interpretable spatio-temporal trajectory prediction framework is proposed for autonomous driving, integrating temporal importance learning and graph-based interaction reasoning to model complex traffic behaviors.
- A comprehensive explainability-driven evaluation approach is introduced to analyze the decision-making behavior of the proposed model through temporal occlusion analysis, interaction sensitivity studies, spatial perturbation experiments, and gradient-based feature attribution methods. These analyses provide insights into how the model captures temporal motion dynamics, neighboring interactions, and environmental context during trajectory forecasting.
- An edge-deployable optimization strategy is developed using mixed-precision inference, adaptive graph simplification, and efficient inference configuration for practical deployment on the resource-constrained NVIDIA Jetson Xavier NX hardware platform and validated through latency, power consumption, memory utilization, and throughput analysis.

Although the individual components employed in the proposed framework have been investigated previously in different contexts, the novelty of this work lies in their unified co-design for deployment-oriented trajectory forecasting rather than in any individual architectural module. Specifically, the proposed framework simultaneously addresses three practical objectives: competitive trajectory forecasting performance, model interpretability through complementary explainability analyses, and experimentally validated embedded deployment on physical edge hardware. To the best of our knowledge, no prior graph-based trajectory forecasting study has jointly reported physical deployment characteristics (latency, power consumption, and thermal behavior) together with temporal, spatial, interaction-based, and gradient-based interpretability analyses for the same deployed model. This integrated evaluation framework therefore represents the principal contribution of the present work.

## 2. Related Works

A spatio-temporal graph approach has become widely used to represent the agent interaction and road topological structure simultaneously. The authors of GSTCN (Graph-based Spatial-Temporal Convolutional Network) [19] suggested incorporating GCN with 1D convolutional encoders and a GRU (Gated Recurrent Unit) decoder for the task of multi-agent trajectory forecasting, considering the NGSIM benchmark. GNN-RNN (Graph Neural Network-Recurrent Neural Network) encoder–decoder architecture [20] uses graph structures of the inter-vehicle interactions for simultaneous multi-agent forecasting using directed graphs. Abdelraouf et al. [21] have used the GCN with LSTM neural networks to make personalization for interaction-aware trajectory forecasting by capturing each driver’s behavior individually. A dynamic heterogeneous graph convolutional recurrent network was developed by Gao et al. [22] for encoding the vehicles and lanes, their temporal dynamics, and spatio-temporal dependency. However, many of these approaches rely primarily on static temporal encoding mechanisms and provide limited analysis regarding the relative behavioral importance of temporal observations during forecasting.

Producing various lane-consistent future trajectories is another vital criterion for realistic self-driving scenarios. Deo et al. introduced a multimodal prediction model based on lane graph traversal, where the predictions are generated conditionally on the possible lane paths [23]. This approach allows structured and map-aware predictions to be obtained. Wang et al. proposed JAL-MTP (Joint Agent and Lane Multimodal Trajectory Prediction) [24], which jointly represents dynamic agent states and static lane segments as instance-level lanes, using Recurrent Lane Attention (RLA) to generate map-adaptive trajectories on the Argoverse benchmark. Kim et al. proposed a two-stage prediction network with Trajectory Prediction Attention (TPA) [25] and a dedicated lane loss function to improve trajectory diversity while maintaining accuracy. Luo et al. presented an attention-based multimodal trajectory prediction model, where standard multi-head attention is replaced by mode-conditioned attention, improving interpretability [26]. Recent lightweight transformer approaches such as SIMPL [27] and state-space forecasting architectures such as Trajectory Mamba [28] have further improved multimodal prediction capability; however, most remain optimized primarily for server-grade GPU execution rather than embedded deployment scenarios.

The lack of interpretability in deep learning-based trajectory prediction models poses significant risks to the safety of self-driving vehicles. Maag et al. introduced a lane change prediction framework [29] using Layer Normalized LSTM with Layer-wise Relevance Propagation (LRP), providing human-understandable explanations on a digital twin platform. Peng et al. proposed LC-LLM, Lane Change Large Language Model [30] for predicting lane change intentions and trajectory by generating human-like language explanations. Kim et al. proposed Traj-Explainer [31], a multimodal trajectory prediction system equipped with explainability models determining how certain features affect the prediction results. Atakishiyev et al. [32] performed extensive research on Explainable AI (XAI) methods applied to decision-making systems in autonomous driving, emphasizing gradient saliency, attention maps, and occlusion sensitivity analysis as vital components to create reliable prediction models. However, limited prior work jointly investigates explainability and deployment-aware trajectory forecasting under embedded resource constraints.

Application of trajectory prediction methods on resource-constrained devices is yet to be extensively investigated. Huang et al. suggested the use of a Recurrent Convolutional Attention network (ReCoAt) [33], which uses a combination of Recurrent Neural Networks (RNNs) for temporal encoding, CNNs for map processing, and distance-based attention mechanisms, trained using the Waymo Open Motion dataset. Wu et al. designed the State Refinement Graph Attention Network (SRGAT) [34], a goal-driven graph attention mechanism that utilizes transformers to capture long-range dependencies and employs a dual-branch multimodal decoder for enhanced performance, tested on the Argoverse and nuScenes datasets. Swaminathan et al. [35] explored the inference capabilities of deep neural networks on NVIDIA Jetson platforms, demonstrating a maximum 7x speedup through FP16 quantization and TensorRT optimization. Paliari et al. [36] provided a review of edge AI deployment, focusing on pruning, quantization, and inference techniques. Gao et al. [37] developed Query-Informed Network QINET, delivering high performance with low complexity. More recently, task-driven computation offloading frameworks, such as the priority-aware deep reinforcement learning approach proposed by Hao et al. [38], offer a complementary strategy for managing latency-critical inference workloads under resource-constrained conditions and represent a promising direction for further reducing embedded inference latency in future extensions of this work. Although these approaches improve forecasting accuracy or computational efficiency, embedded deployment validation involving latency, thermal behavior, and power-aware execution on physical autonomous driving hardware remains insufficiently explored.

As surveyed above, existing trajectory forecasting studies can generally be categorized into three directions: accuracy-oriented methods emphasizing low displacement error through computationally intensive architectures; edge-aware approaches focusing primarily on lightweight inference efficiency; and explainability-driven methods investigating model interpretability. However, limited prior work jointly investigates temporal behavior-aware forecasting, lane-aware graph interaction reasoning, embedded edge deployment validation, and multi-dimensional interpretability within a unified framework. The present work specifically targets this intersection by investigating interpretable and deployment-aware trajectory forecasting under embedded autonomous driving constraints.

## 3. Design and Implementation

**Figure 1:**
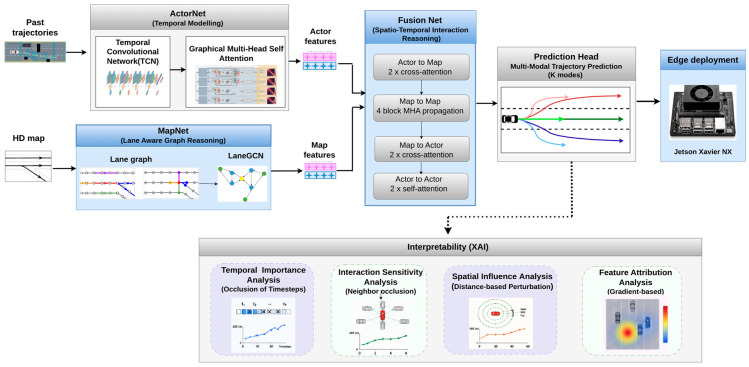
Overall architecture of the proposed interpretable and edge-deployable spatio-temporal trajectory prediction approach. Solid arrows indicate the primary data flow through the prediction pipeline, whereas dotted arrows denote the interpretability (XAI) analysis. Different colors are used only to distinguish functional modules and improve the readability of the architecture.

The model architecture for the proposed approach is shown in Figure 1. It shows the overall design of the network architecture that consists of four main modules, namely ActorNet, MapNet, FusionNet, and Prediction Head.

### 3.1. ActorNet

The main purpose of ActorNet is to learn the motion pattern from past trajectories. In this work, the past trajectory history of 20 frames is used to predict the future trajectory of the agent over the next 30 timesteps (3 s). ActorNet is designed in such a way as to learn the motion pattern of the ego vehicle. The observed motion history can be represented as follows.

(1) $$X={\left\{\left(dx_{t,}dy_{t,}v_{t}\right)\right\}}_{t=1}^{T}$$

where $dx_{t}$, $dy_{t}$ are the relative displacements of the vehicle between the consecutive frames and $v_{t}$ is the vehicle velocity at time ‘t’. The sequence contains ‘T’ past observations, and in our work T 20. ActorNet captures how a vehicle moves in space and how its speed changes over time; this representation is very important for understanding driving behavior.

ActorNet has two main parts: a Temporal Convolutional Network (TCN) that recognizes temporal motion patterns, and a Multi-Head Self-Attention (MHSA) that finds the most informative features. This allows for the learning of short-term as well as long-term dependencies. Lastly, there is a pooling operation that aggregates all the information from all timesteps to a single vector.

#### 3.1.1. Temporal Convolution Module (TCN)

**Figure 2:**
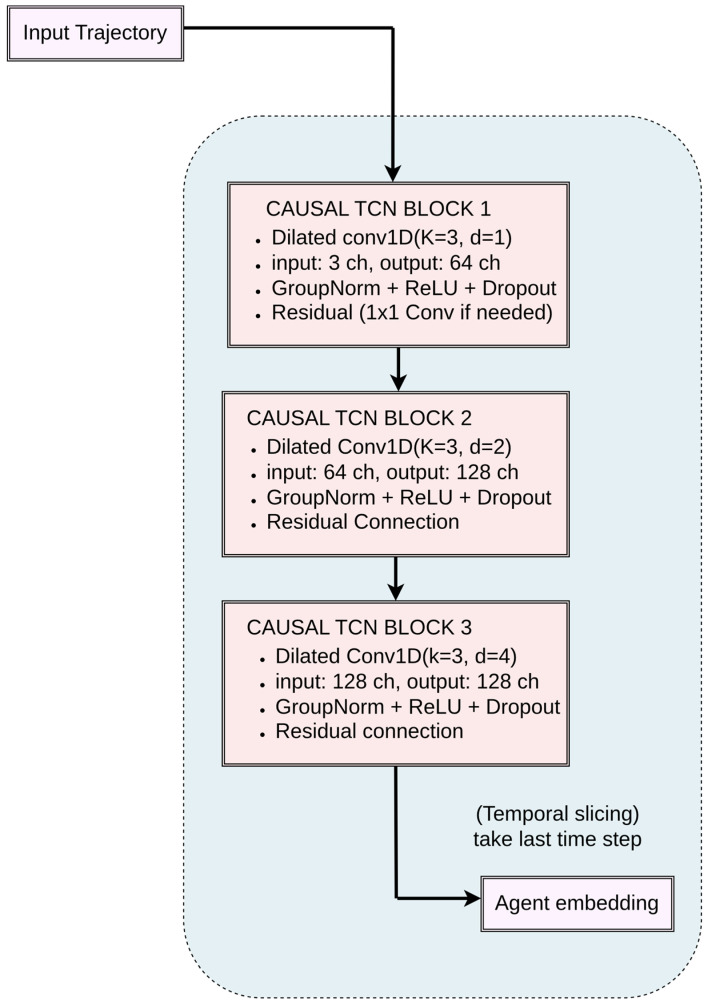
Architecture of the Causal TCN Block. Solid arrows indicate the forward data flow through successive Causal TCN blocks. Different colors are used only to distinguish functional components and improve the readability of the architecture.

The main purpose of TCN in ActorNet is to extract meaningful temporal patterns from the motion sequence of the agent’s past. It analyzes the entire sequence in parallel using a 1D CNN, which makes training faster and more stable. The main advantage of using TCN is that a causal TCN ensures that the predictions at timestep *t* only depend on the current and past observations and not on future information. So, it matches the real-world condition where the future motion is unknown. Figure 2 shows the architecture of the TCN block.

Given the input sequence X, a dilated temporal convolution is defined as

(2) $$H_{t}=\sigma \left(\sum_{k=0}^{k-1}w_{k}\;.\;X_{t-rk}\right)$$

where k is the kernel size, r is the dilation factor, $w_{k}$ are convolution weights, and σ(·) is the ReLU activation function. Dilation in TCN helps allow the convolution to skip timesteps, which helps increase the temporal receptive field without increasing the number of parameters. This allows the model to analyze long motion history using fewer layers and parameters. When predicting trajectories, it is crucial to capture long-term temporal dependencies since movement patterns tend to develop slowly. In other words, long-term temporal dependencies help identify any slight movement trend such as lateral drifting. A residual connection is also added in the block to provide proper gradient flow. The output at this stage can be represented as

(3) $$H_{out}=H_{TCN}+H_{res}$$

where $H_{TCN}$ denotes the output of TCN and $H_{res}$ represents the output of the residual connection. This layer helps to learn without losing low-level motion details.

The motion trajectory passes through the processing of three causal dilated convolution layers with increasing dilation rates (d = 1, 2, 4) for capturing short-term, mid-term, and long-term dependencies. The layers include a combination of Conv1D, Group Normalization, ReLU, Dropout, and residual connection for stable learning. The last timestep features are extracted using temporal slicing, which is a compact form of the vehicle’s motion history.

#### 3.1.2. Multi-Head Self-Attention (MHSA)

Temporal Convolutional Networks are effective models for learning short-term and long-term motion patterns, but they process all past timesteps in a uniform manner. In real-world driving scenarios, subtle changes in motion, such as steering adjustments or lateral drift, occur only at specific timesteps, and these changes can serve as early indicators of deviation from the trajectory. To identify and emphasize such important trends, a Multi-Head Self-Attention Module is applied after the TCN output.

Let the TCN output be represented as

(4) $$H_{TCN}\in \;R^{T\times C}$$

where T is the number of observed timesteps and C is the feature dimension. The attention mechanism projects temporal features into three different spaces called query (Q), key (k), and value (V).

(5) $$Q=H_{TCN}W_{Q,}K=H_{TCN}W_{K,}V=H_{TCN}W_{V}$$

where $W_{Q}\in R^{C\times d}$, $W_{K}\in R^{C\times d}$ and $W_{V}\in R^{C\times d}$ are learnable projection matrices and d is the attention embedding dimension. These matrices help to learn different representations of the same temporal features, and they allow the model to compare every timestep. Attention score can be computed by

(6) $$Attn\left(Q,K,V\right)=\mathrm{soft}\;\max\left(\frac{QK^{T}}{\sqrt{C}}\right)V$$

**Figure 3:**
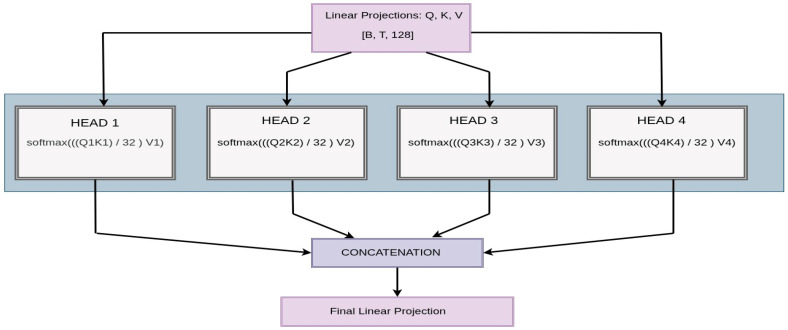
Architecture of the Multi-Head Self-Attention (MHSA) Module. Solid arrows indicate the flow of information through the attention mechanism. Different colors are used only to distinguish functional components and improve the readability of the architecture.

This process involves all the timesteps’ relationships, where more weight is given to more informative ones for better prediction of behavior. In this paper, four attention heads are used to focus on important motion patterns such as lateral movement or steering changes, as presented in the MHSA structure of ActorNet. Figure 3 shows the overall structure of Multi-Headed Self-Attention (MHSA) in ActorNet, where the input temporal features are linearly projected into queries (Q), keys (K), and values (V), and then processed by multiple parallel attention heads. In each head, the scaled dot-product attention is calculated to focus on various temporal dependencies and highlight key motion patterns. The results of all heads are then combined through a final linear projection to get a refined representation of the temporal features.

(7) $$H_{attn}=concat\left(head_{1},\ldots \ldots \ldots ..,head_{n}\right)w_{0}$$

where n is the number of attention heads, and $\;w_{0}$ is the output projection matrix. After this, average pooling is performed across temporal dimensions. It helps to summarize the vehicle’s entire motion history. An average pooling operation is performed, $F_{factor}=pooling\left(H_{attn}\right)$. Here, it aggregates the attention-weighted temporal features into a compact embedding. The resulting actor feature embedding captures detailed temporal dynamics.

### 3.2. MapNet

ActorNet explains the motion trends of the vehicle, but it does not provide information regarding the road geometry. But accurate trajectory prediction behavior strongly depends on the lane structure, lane direction, and connectivity. So here, this MapNet module is used to model the road geometry and lane connectivity using HD map information. HD map can be represented as

(8) $$g=\left(V,E\right)$$

where V represents a lane centerline segment and E represents lane connections such as predecessor, successor, left-adjacent, and right-adjacent lanes. By integrating this map structure, the model captures lane connectivity and understands how these relationships influence the vehicle’s motion behavior. Using these connections, we are forming a lane graph where each segment of the lane is a node, and the interaction between the lanes becomes the edges. Finally, to get meaningful lane features, a graph attention mechanism known as LaneGAT is applied. For each node V, information is collected from its neighboring lanes U using attention-based aggregation, where $U\in N\left(V\right)$ and $N\left(V\right)$ denotes the set of all nodes directly connected to V. The lane feature after aggregating attention weights from its neighboring lane nodes through can be calculated by

(9) $$h_{v}^{\prime }=\sum_{\;u\in N\left(v\right)}\alpha_{vu}\phi \left(h_{u}\right)$$

where h_u_ is the feature of the neighboring lane u, $\phi \left(.\right)$ is a learnable transformation function, and $\alpha_{vu}$ is an attention weight that indicates how relevant lane u is to lane v. More weight is given to lanes that are more aligned with the vehicle’s current motion or more likely to be influential in future maneuvers. The final output from MapNet can be represented as $F_{map}$. It captures geometric and topological lane information. It is then passed to the next stage, where the outputs from ActorNet and MapNet are combined.

### 3.3. FusionNet

FusionNet integrates motion features extracted from ActorNet and lane features extracted from MapNet to enable the model to comprehend how the motion of a vehicle relates to the road. FusionNet has three sequential interactions: Actor-to-Map (A2M), Map-to-Map (M2M), and Map-to-Actor (M2A), in which information flows from vehicles to lanes, then to connected lanes, and finally back to vehicles.

#### 3.3.1. Actor-to-Map

In the Actor-to-Map interaction, lane features are updated based on information received from the surrounding vehicles. Since trajectory prediction depends on both motion history and road structure, the lane features are updated with dynamic traffic information. Information about turn directions, traffic signals, and intersection characteristics is also added to the lane features, which means the lane features are not only geometric but also traffic-aware.

Let $h_{v}$ be the original feature of the lane v and $m_{v}$ be its semantic attribute vector. The enriched lane feature can be computed as

(10) $$\hat{h_{v}}=f_{meta}\left(\left[h_{v}∥m_{v}\right]\right)$$

where $f_{meta}\left(.\right)$ represents a lightweight neural transformation model that consists of linear projection, normalization, and non-linear activation. For each lane, only nearby vehicles whose distance is within a pre-defined threshold d are used for the interaction process. The set of associated vehicles $A\left(v\right)$ can be defined as

(11) $$A\left(v\right)=\left\{i\left|∥c_{i}-c_{v}∥⋜d\right|\right\}$$

where ‘i’ is the index of vehicles in those scenes, ‘$c_{i}$’ is the actor center position and $c_{v}$ is the lane center position. For each nearby vehicle, a relevance score is computed as

(12) $$e_{iv}=f_{att}\left(a_{i\;,}\hat{h_{v}}\right)$$

where $e_{iv}$ is a raw importance score, $a_{i}$ is the vector representation of vehicle *i*’s motion behavior, and $\hat{h_{v}}$ is the lane feature from the previous step.

In the next step, the compatibility scores between the lane and the nearby vehicles are converted to normalized attention weights through a softmax function. The attention weight of vehicle i with respect to lane v at the l-th i refinement layer $\alpha_{iv}^{\left(l\right)}$ is given by

(13) $$\alpha_{iv}^{\left(l\right)}=\frac{\exp\left(e_{iv}^{\left(l\right)}\right)}{\sum_{k\in A\left(v\right)}\exp\left(e_{kv}^{\left(l\right)}\right)}$$

where $e_{iv}$ represents the compatibility score between vehicle i and lane, computed in the previous step, and Equation (13) explains how each vehicle influences the lane representation. Here $\sum_{k\in A\left(v\right)}\exp\left(e_{kv}^{\left(l\right)}\right)$ acts as a normalization term that converts raw compatibility scores into a probability distribution across all nearby vehicles. Finally, the lane feature is updated by aggregating weighted vehicle contributions from nearby vehicles calculated as

(14) $$h_{v}^{A2M}=\sum_{\;i\in A\left(v\right)}\alpha_{iv}a_{i}$$

where $h_{v}^{A2M}$ denotes the updated feature of lane v after Actor-to-Map interaction. This representation integrates both static lane properties and dynamic traffic information from nearby vehicles.

#### 3.3.2. Map-to-Map Interaction

After the lane features are updated with the traffic information using the A2M interaction, the Map-to-Map interaction is used to share the information across the connected lanes. The structure of the roads is connected; hence, the state of the traffic on a particular lane is related to its preceding, succeeding, and adjacent lanes. Using the Map-to-Map interaction, every lane is able to grasp the context beyond its immediate environment.

A lane only interacts with its directly connected lanes:

(15) $$N\left(v\right)=\left\{predecessor,successor,left,right\;lane\;of\;v\right\}$$

where predecessor/successor lanes capture longitudinal connectivity and left/right lanes capture lateral relationships. For each neighboring lane $u\in N\left(v\right)$, a relevance score is computed as,

(16) $$e_{vu}=f_{lane}\left(h_{v}^{A2M},h_{u}^{A2M}\right)$$

where $h_{v}^{A2M}$ be the traffic-aware feature of lane v obtained from the A2M stage and $h_{u}^{A2M}$ represents the traffic-aware feature representations of neighbor lanes u, obtained after the Actor-to-Map interaction. The function $f_{lane}\left(.\right)$ is a learnable scoring function implemented as a lightweight neural network that models the compatibility between two connected lane segments.

These scores are normalized using softmax to obtain attention weights:

(17) $$\alpha_{vu}=\frac{\exp\left(e_{vu}\right)}{\sum_{k\in N\left(v\right)}\exp\left(e_{vk}\right)}$$

The attention weights that are applied to all the adjacent lanes use the softmax operation, which means that the sum of all the weights is equal to 1. This mechanism allows adjacent lanes to interact based on their relative importance when updating the lane v. Adjacent lanes with higher relevance scores contribute more significantly to the lane representation update, while those with lower relevance scores have a reduced influence.

Using these attention weights, the lane representation is updated by aggregating information from connected lanes as

(18) $$h_{v}^{M2M}=\sum_{\;U\in N\left(v\right)}\alpha_{vu}h_{u}^{A2M}$$

where $h_{v}^{M2M}$ represents the traffic-aware and context-enriched feature of the neighboring lane v, which is achieved via Actor-Map interaction. By using attention-weighted aggregation, each lane is able to receive information from the connected lanes based on their structural and traffic relevance.

#### 3.3.3. Map-to-Actor Interaction

After the Map-to-Map interaction, there are representations of each lane that are rich, incorporating both traffic and road structure. The final step is to incorporate this rich lane information back into each vehicle’s representation, effectively making actors lane-aware before predicting their trajectories. This is done through the Map-to-Actor interaction.

For each actor *i*, only spatially nearby lanes are considered, filtered by a distance threshold *d*. Let $a_{i}$ denote the feature representation of actor i, obtained from the ActorNet, and let $h_{v}^{M2M}$ denote the refined feature of lane v after the Map-to-Map interaction. For each actor, only spatially relevant lanes are considered during the interaction. The set of candidate lanes for actor i is defined as

(19) $$N\left(i\right)=\left\{v\left|∥c_{v}-c_{i}\right|∥⋜d\right\}$$

where $c_{i}$ and $c_{v}$ represent the locations of the actor i and lane v, respectively. d is the distance threshold. This ensures that the vehicle only interacts with lanes that it can occupy or move toward. For each lane $v\in N\left(i\right)$, a relevance score is computed to measure how informative that lane is for guiding the motion of actor i:

(20) $$e_{vi}=f_{M2A}\left(h_{v}^{M2M},a_{i}\right)$$

where $f_{M2A}\left(.\right)$ is a lightweight learnable scoring function used to compute the compatibility between lane information and the actor’s current state of motion. This scoring function uses a softmax normalization.

For an actor i, with candidate lanes $v\in N\left(i\right)$, the attention weight is calculated as

(21) $$\gamma_{vi}=\frac{\exp\left(e_{vi}\right)}{\sum_{\;k\in N\left(i\right)}\exp\left(e_{ki}\right)}$$

Let $a_{i}^{M2A}$ denote the map-aware feature of actor *i* obtained after the Map-to-Actor interaction. Using these attention weights, the actor feature is updated by aggregating information from the refined lane representations:

(22) $$a_{i}^{M2A}=\sum_{\;v\in N\left(i\right)}\gamma_{vi}h_{v}^{M2M}$$

Aggregation allows the actor to incorporate the representations in the lanes, making its representation similar to the structure of the road.

#### 3.3.4. Actor to Actor

Although the Map-to-Actor interaction provides each vehicle with lane awareness, it does not account for the influence of the surrounding vehicles on the movement of each vehicle. The Actor-to-Actor interaction provides a solution for this problem by enabling each vehicle to aggregate the movement context from the surrounding vehicles. This is a critical component for modeling behaviors such as car-following, merging, and lane-changing based on the surrounding traffic.

For each actor i, only nearby actors are considered during interaction. The neighborhood of actor i is defined as

(23) $$N\left(i\right)=\left\{j\left|∥c_{j}-c_{i}∥⋜d_{a}\right|\right\}$$

where $c_{i}$ and $c_{j}$ denote the spatial position of actors i and j, respectively, and d is a pre-defined threshold.

For each neighboring actor $j\in N\left(i\right)$, a relevance score is computed to quantify how strongly the motion of actor J influences the future behavior of actor i:

(24) $$e_{ij}=f_{a2a}\left(a_{i}^{M2A},a_{j}^{M2A}\right)$$

Here $a_{i}^{M2A}$ and $a_{j}^{M2A}$ denote the map-aware feature representations of actors i and j, respectively. $F\left(.\right)$ is a neural network capable of learning an actor’s compatibility and serves to calculate how much two different people interact based on how their movement states correspond to each other.

After computing the relevance score between actor i and each neighboring actor j, these scores are normalized to obtain attention weights. The attention weight assigned to actor j with respect to actor *i* is computed as

(25) $$\alpha_{ij}=\frac{\exp\left(e_{ij}\right)}{\sum_{\;k\in N\left(i\right)}\exp\left(e_{ik}\right)}$$

Here, $e_{ij}$ denotes the relevance score measuring how strongly actor j influences actor i, and $N\left(i\right)$ represents the set of neighboring actors around actor i.

Using the normalized attention weights, the actor feature is updated by aggregating information from neighboring actors:

(26) $$a_{i}^{A2A}=\sum_{\;j\in N\left(i\right)}\alpha_{ij}a_{j}^{M2A}$$

In this case, $a_{j}^{M2A}$ represents the map-aware attribute of the neighboring actor j after the Map-to-Actor interaction. In addition, the attention weight $\alpha_{ij}$ determines the level of influence actor j imparts to the new representation of actor i. Overall, this represents a combination of a vehicle’s motion history, road structure, and social interactions with nearby vehicles. This allows for accurate forecasting of vehicle trajectories as well as complex driving scenarios in a dense environment.

### 3.4. Prediction Head of Multimodal Trajectory

After the Actor-Map Fusion step, every actor i is described by a fused feature vector $z_{i}\in R^{d}$ that combines the temporal dynamics of motion, the geometry of the lanes, and the traffic context. The fused feature vector $z_{i}$ is then fed into the Prediction Head to predict the outputs related to the future motion and behavior.

Based on the fused representation $z_{i}$, the model predicts the future trajectory of the ego vehicle over a fixed prediction horizon of H timesteps. Instead of predicting a single deterministic trajectory, the model predicts multiple plausible future trajectories to capture the inherent uncertainty in driving behavior. The k-th predicted trajectory for actor is defined as

(27) $$\hat{y_{i}^{\left(k\right)}}={\left\{\left(\hat{x_{i,t}^{\left(k\right)}},\hat{y_{i,t}^{\left(k\right)}}\right)\right\}}_{t=1}^{H}\;K\in \left\{\left(1,\ldots K\right)\right\}$$

where $\left(\hat{x_{i,t}^{\left(k\right)}},\hat{x_{i,t}^{\left(k\right)}}\right)$ denotes the predicted 2D position at timestep *t*, and *k* represents the number of trajectory modes. Each trajectory mode is obtained through a regression function applied to the fused feature:

(28) $$\hat{y_{i}^{\left(k\right)}}=f_{traj}^{\left(k\right)}\left(z_{i}\right)$$

This multimodal representation allows the model to capture various possible driving intentions, such as lane keeping and lane changing, in a unified manner.

### 3.5. Mode Probability Estimation

In addition to the trajectory generation, the Prediction Head also predicts the confidence of each trajectory mode. The classification branch predicts the mode probabilities as

(29) $$P_{i}^{\left(k\right)}=\mathrm{soft}\;\max\left(f_{cls}\left(z_{i}\right)\right)$$

where $P_{i}^{\left(k\right)}$ represents the likelihood of the k-th trajectory mode. This is useful for the model to rank the predicted trajectories according to their plausibility, such that more realistic motion patterns are assigned to higher confidence.

### 3.6. Best of k Trajectory Selection

During evaluation, all predicted trajectories are compared with the ground truth future trajectory

(30) $$Y_{i}={\left\{\left(x_{i,t},\;y_{i,t}\right)\right\}}_{t=1}^{H}$$

The trajectory with the minimum prediction error is selected as the best prediction:

(31) $$K^{*}=\arg\;M\;in\;Error\left(\hat{y^{\left(k\right)}},y_{i}\right)$$

### 3.7. Evaluation Metrics

For the quantitative analysis of prediction accuracy, standard metrics for trajectory forecasting are used. The Average Displacement Error (ADE) computes the average Euclidean distance between the predicted and ground truth trajectories over the entire prediction horizon

(32) $$ADE=\frac{1}{H}\sum_{\;t=1}^{\;H}{\left|\left|\left(\hat{x_{t}},\hat{y_{t}}\right)-\left(x_{t},y_{t}\right)\right|\right|}_{2}$$

The Final Displacement Error (FDE) evaluates the prediction accuracy at the final timestep:

(33) $$FDE={\left|\left|\left(\hat{x_{H}},\hat{y_{H}}\right)-\left(x_{H},y_{H}\right)\right|\right|}_{2}$$

In the multimodal setting, the minimum error across all trajectory modes is reported as minADE and minFDE:

(34) $$\min ADE=\underset{k\in \left\{1,\ldots .,k\right\}}{\min}ADE^{\left(k\right)},\min FDE=\underset{k\in \left\{1,\ldots ..k\right\}}{\min}FDE$$

Miss Rate (MR) is used to evaluate the failure of the prediction when the error in the end displacement is greater than a certain threshold value δ.

Miss Rate (MR) assesses the percentage of trajectory estimates for which the Final Displacement Error surpasses a pre-defined limit δ, representing cases where the estimated trajectory diverges considerably from the actual trajectory:

(35) $$MR=\frac{1}{N}\sum_{i=1}^{N}I\left(FDE_{i}>\delta \right)$$

where N is the total number of trajectory predictions, and ${\mathrm{FDE}}_{i}s$ is the Final Displacement Error of the i-th trajectory prediction, and $I\left(.\right)$ is the indicator function that returns 1 if the condition is true and 0 otherwise.

Drivable Area Compliance (DAC): This is a metric used to calculate the percentage of the trajectories predicted by a model that stay inside the valid driven area:

(36) $$DAC=\frac{1}{N}\sum_{i=1}^{N}I\left(\hat{Y_{i}}\right)\;\subseteq D$$

where $\hat{Y_{i}}$ denotes the predicted trajectory for the i-th sample, and D represents the drivable area defined by the HD map.

## 4. Experiments and Results

### 4.1. Dataset Description

The model is tested on the Argoverse-1 Motion Forecasting Dataset, which is extensively utilized as a benchmark in trajectory prediction tasks. The Argoverse-1 motion forecasting dataset consists of 323,557 traffic scenarios from Pittsburgh and Miami that involve various driving behaviors, including lane change, merging, and intersection scenarios. Each trajectory segment is recorded for 5 s with a frame rate of 10 Hz, yielding 50 timesteps per scene. In the present work, an actor is defined as any dynamic traffic agent that impacts trajectory prediction, which includes the focal actor and other agents. The proposed model learns from the first 2 s (20 timesteps) and forecasts for the next 3 s (30 timesteps). An actor’s historical trajectory is encoded as a sequence of 2D displacement vectors in an ego-centric coordinate system, where the focal actor is centered at the origin and aligned with the positive *x*-axis, enabling trajectory prediction that is invariant to global translations.

In each scene, an HD map is provided which contains geometric and topological structure information about the road network. HD maps include the geometry of lane centerlines represented as ordered polylines, and also connectivity of lanes, which include predecessors, successors, left adjacent lanes, and right adjacent lanes. These components are then used to generate vectorized maps as opposed to rasters, which preserve the geometrical relations between roads and objects/vehicles. The lane graph generated from this, having nodes for lane centerline segments and edges representing their topological connectivity, is then used as input for the MapNet module. Each scene, on average, has 19.4 actors in it, making it highly suitable for testing motion planning models which need to perform reasoning based on trajectories and the influence of other agents on one another.

### 4.2. Implementation Details

The proposed model is implemented using PyTorch (version 2.7.1+cu118) and trained with the Adam optimizer and an initial learning rate of 1 × 10^−3^, which is decayed to 1 × 10^−4^ after 40 epochs with a step learning rate scheduler. The model is trained for 45 epochs with display and validation iterations of 60,000 and 80,000, respectively. The number of data loading workers is set to 0 to provide stable processing of graph-structured inputs. The experiments are carried out with two NVIDIA A100 Tensor Core GPUs with Ampere architecture, which support high memory bandwidth and efficient computation for attention and graph operations. The model takes 20 past frames and predicts 30 future frames with six trajectory modes (K = 6). All experiments are performed in the BEV coordinate system with the target agent at the origin, and the same configuration and hyperparameters are used for all experiments.

The training process takes place on a workstation that features two NVIDIA A100 Tensor Core GPUs. As a result, operations such as graph convolution, temporal convolution, and attention can be executed effectively due to the high memory bandwidth and parallel processing capabilities of the hardware. To evaluate the performance in the deployment phase, inference runs are also implemented using the NVIDIA Jetson Xavier NX Developer Kit (manufactured by NVIDIA Corporation, Santa Clara, CA, USA), which works in 10 W desktop mode and can be considered as an edge-computing platform for self-driving vehicles. The monitoring results provided by jtop show stable resource consumption, a relatively low operating temperature (about 58.9 °C), minimal GPU memory usage (around 16.8 MB), and efficient power usage.

### 4.3. Quantitative Performance Evaluation of the Proposed Model

Quantitative performance of the suggested framework is provided in Table 1. In particular, the minADE and minFDE obtained by the suggested framework are 0.90 m and 1.50 m, respectively, demonstrating highly accurate trajectory prediction. Miss Rate (MR) of 0.1941 suggests that the suggested model performs quite well in terms of avoiding failures in generating a plausible trajectory for vehicles. Finally, the Drivable Area Compliance (DAC) score of 0.95 proves that most of the generated trajectories stay within the drivable areas of the road, thus proving the feasibility of generating physically realistic and drivable road trajectory predictions. All the above results prove that the proposed method is successful in incorporating the dynamics of motion with lane-aware contextual information.

### 4.4. Comparison with Transformer-Based Methods on Argoverse 1

Comparison in Table 2 illustrates the comparison between the proposed hybrid approach and recent trajectory prediction approaches based on transformers on the Argoverse 1 dataset. The Scene Transformer [14], which is a unified model where all agents and timesteps are modeled by applying full spatio-temporal self-attention, together with mmTransformer [39], ADAPT [40], SIMPL [27], and HiVT [12], shows better performance with regard to the displacement error metric. However, these architectures are limited in terms of scalability, making them non-applicable at the edge level. Both the Scene Transformer, where joint self-attention is applied to all agents and timesteps, and mmTransformer and HiVT, which model interaction among several agents, show a high complexity of O(A^2^·T) that scales quadratically with the density of agents in a scene. The proposed ActorNet, which makes use of MHSA only in the temporal dimension (T = 20) for each agent, is of O(T^2^) complexity.

The proposed model obtains a minADE of 0.90 m, minFDE of 1.50 m, and Miss Rate of 0.19, thus exhibiting moderate accuracy performance that is adequate to fulfill the needs of structured urban scenarios, where the trajectory feasibility and adherence to lanes are more important for safety than any additional improvements over the transformer-based approach. It is worth noting that none of the benchmarked methods were tested on embedded devices and are prone to memory consumption due to extensive use of softmax-based attention mechanisms between multiple agents in the model and high parameter counts. On the other hand, the proposed model has been successfully implemented on the NVIDIA Jetson with a frame rate of 7.95 samples/s, utilizing no more than 12.86 W of power, without using any model compression algorithm, implying that the designed architecture is efficient by its nature. Thus, the accuracy–performance–compliance trade-off can be stated regarding all benchmarked models except the proposed one.

It is acknowledged that several recent forecasting architectures have further advanced trajectory prediction accuracy on the Argoverse benchmark. Notable among these is SIMPL [27], which achieves a minADE of 0.66 m with a relatively lightweight parameter count of 2.65 M. More recent approaches such as Trajectory Mamba [28], which utilizes selective state-space sequence modeling for trajectory forecasting, report further improvements in displacement accuracy. However, these methods are primarily designed and evaluated on server-grade GPU platforms and currently provide limited analysis regarding embedded deployment feasibility, thermal behavior, latency stability, or power-aware execution on edge hardware. In contrast, the primary objective of the proposed framework is not solely to optimize forecasting accuracy, but to investigate an interpretable and deployment-aware operating point for graph-based trajectory prediction under embedded resource constraints. To the best of our knowledge, this study is among the few trajectory forecasting works to provide measured latency, power consumption, thermal characteristics, and resource utilization analysis on a physical NVIDIA Jetson embedded platform while maintaining reliable forecasting performance.

**Figure 4:**
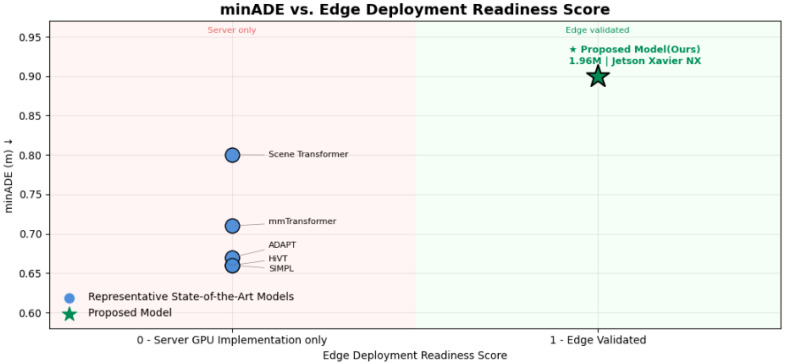
Comparison of trajectory forecasting accuracy (minADE) and edge deployment readiness across recent forecasting architectures. The downward arrow (↓) indicates that lower minADE values correspond to better forecasting accuracy, while the Edge Deployment Readiness Score distinguishes server-only implementations (0) from edge-validated models (1).

Figure 4 illustrates the relationship between forecasting accuracy and deployment readiness across recent trajectory prediction architectures. The comparison highlights that the present work targets a deployment-aware operating point by balancing forecasting performance with practical embedded execution feasibility under resource-constrained autonomous driving conditions.

Although the proposed framework does not achieve the lowest displacement error reported on the Argoverse benchmark, the observed performance gap should be interpreted in the context of the design objectives of this work. Compared with recent transformer-based methods such as HiVT and SIMPL (minADE = 0.66 m), the proposed framework exhibits an absolute increase of 0.24 m (approximately 26.7%) in minimum Average Displacement Error. This difference is acknowledged and represents a deliberate trade-off rather than an unintended limitation. As demonstrated by the additional embedded deployment experiments presented in Section 4.5, the reduction in forecasting accuracy is accompanied by measurable improvements in deployment efficiency, including lower inference latency, higher effective throughput, and more balanced computational resource utilization on the NVIDIA Jetson Xavier NX platform. Consequently, the proposed framework should be viewed as targeting a deployment-oriented operating point rather than maximizing displacement accuracy alone.

### 4.5. Edge Deployment Evaluation on NVIDIA Jetson

To the best of our knowledge, this is among the few works that evaluate trajectory prediction models on embedded edge platforms. Due to the dynamic graph operations inherent in LaneGCN, conventional optimization techniques such as TensorRT (Tensor Runtime) and INT8 (8-bit Integer) quantization are not directly applicable. To verify the efficiency of the proposed trajectory prediction method, the entire framework was executed and evaluated using the NVIDIA Jetson platform—an example of edge computing. There were several deployment-aware optimizations employed during inference, including FP16 mixed-precision execution with Automatic Mixed Precision (AMP), adaptive lane and actor selection for graph simplification, temporal downsampling for reducing redundant sequential computations, optimized batch processing for improved GPU utilization, and data loading optimization techniques such as pinned memory and prefetching to reduce data transfer overhead. In this manner, the latency, computational speed, resource usage, and power consumption of the device were evaluated. The edge deployment evaluation results of the proposed framework on the NVIDIA Jetson platform are presented in Table 3.

Unlike conventional deployment studies that primarily focus on inference acceleration alone, the proposed framework adopts a deployment-aware optimization strategy specifically tailored for graph-based trajectory forecasting architectures. Quantization-Aware Training (QAT) and INT8 acceleration were investigated during deployment optimization. However, the dynamic graph construction, variable-sized interaction neighborhoods, and irregular message-passing operations inherent in lane-aware graph reasoning introduce substantial challenges for static quantization pipelines and DLA-compatible execution. In particular, dynamic graph indexing and attention-based interaction propagation are not efficiently supported by current TensorRT INT8 optimization workflows designed primarily for static convolutional architectures. Therefore, the proposed deployment strategy focuses on graph simplification through adaptive lane and actor selection, temporal downsampling to reduce redundant sequential processing, and mixed-precision inference for efficient resource utilization. These optimizations collectively reduce graph computation overhead while preserving behavior-critical interaction information required for trajectory prediction in autonomous driving scenarios.

The results of the deployment shown in Table 3 illustrate that the suggested approach can be considered practically feasible to perform trajectory prediction in an effective way on the NVIDIA Jetson embedded platform. In this regard, the suggested approach is capable of delivering an average inference time of 125.74 ms/batch and throughput of 1.99 batches/sec. Moreover, the effective throughput of 7.95 samples/sec suggests a responsive inference rate appropriate for embedded systems deployment. Regarding the resource usage of the suggested approach, it is possible to notice that there is an equal distribution of the workload across hardware components, with CPU usage of 57.34%, GPU usage of 55.88%, and RAM usage of 71.43%. Finally, it should be noted that the average operation temperature of 58.92 °C and power usage of 12.86 W provide evidence for stable and energy-efficient operation.

Further assessments of thermal energy and power consumption have shown that the suggested model can be successfully used in edge-based settings. In particular, the temperature of the edge device stayed within a safe range at 58.92 °C without reaching the threshold of thermal throttling on the Jetson platform, and the power consumption did not exceed 12.86 W. The proposed approach results demonstrate a practical balance between forecasting accuracy, interpretability, deployment efficiency, and resource utilization, making it suitable for edge-aware autonomous driving applications.

While the achieved latency did not yet satisfy the stringent update requirements of tightly coupled Level-4 autonomous driving control loops, it is important to note that trajectory forecasting is generally performed as part of the higher-level planning pipeline rather than the low-level vehicle control loop, which typically operates at substantially higher update frequencies. Consequently, the proposed framework remains suitable for planning-oriented applications such as maneuver anticipation, lane-change prediction, and urban driving assistance, where prediction updates are less time-critical than steering or braking control. Nevertheless, further latency reduction remains an important objective for future deployment. Promising optimization strategies include structured pruning and knowledge distillation of the temporal modeling components, static pre-compilation of fixed-topology lane-graph structures, and dynamic computation-offloading strategies such as those proposed by Hao et al. [38], which may further improve embedded inference efficiency under resource-constrained conditions.

**Figure 5:**
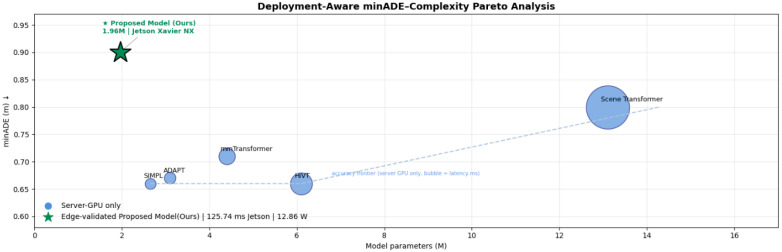
Deployment-aware minADE–complexity Pareto analysis across recent trajectory forecasting architectures. The downward arrow (↓) indicates that lower minADE values correspond to better forecasting accuracy and the x-axis represents model complexity (in million parameters).

The deployment-aware minADE–complexity Pareto analysis shown in Figure 5 demonstrates the tradeoff between forecasting performance and model complexity across recent trajectory prediction methods. While large transformer-based architectures achieve strong displacement accuracy, they typically involve substantially higher parameter complexity and lack validated embedded deployment analysis. In comparison, the proposed framework maintains relatively low model complexity together with experimentally validated edge execution on the NVIDIA Jetson Xavier NX platform.

#### Comparative Edge Deployment Analysis with Recent Trajectory Prediction Models

To further validate the deployment efficiency of the proposed framework, two representative transformer-based trajectory prediction models, namely HiVT [12] and SIMPL [27], were implemented and evaluated on the same NVIDIA Jetson Xavier NX platform using identical deployment settings. The comparative results are presented in Table 4.

Although HiVT and SIMPL represent recent state-of-the-art trajectory prediction methods with superior forecasting accuracy on the Argoverse benchmark, their deployment on the embedded platform incurs noticeably higher computational overhead. HiVT increases the inference latency from 125.73 ms to 151.09 ms, together with higher GPU utilization (65.79%), memory usage (82.41%), and operating temperature (69.46 °C), reflecting the increased computational complexity associated with hierarchical transformer-based processing. SIMPL exhibits the highest inference latency (187.60 ms) and CPU utilization (94.18%), indicating greater dependence on host-side computation during inference.

By comparison, the proposed framework achieves the lowest inference latency (125.73 ms), highest effective throughput (7.95 samples/s), and the most balanced utilization of CPU, GPU, memory, thermal behavior, and power consumption among the evaluated models. These results demonstrate that the proposed architecture provides a more favorable balance between forecasting capability and deployment efficiency for resource-constrained embedded platforms. While transformer-based approaches continue to achieve lower displacement errors, the proposed framework substantially reduces the computational burden required for practical embedded deployment, supporting the deployment-oriented design objective of this work. These comparative experiments directly address the practical deployment gap identified in existing trajectory prediction literature, where embedded hardware evaluations of recent transformer-based forecasting models remain limited.

### 4.6. Deployment Evaluation Across Different Computing Platforms

To complement the Jetson Xavier NX deployment evaluation presented in the previous section, the proposed framework was additionally evaluated on two representative CPU-based platforms, namely an Intel i7-1260P and an Intel Celeron processor (Table 5). The purpose of this comparison is not to benchmark hardware platforms themselves, but to examine whether the deployment characteristics of the proposed trajectory prediction framework remain consistent across different computational architectures. Since the objective of this work is deployment-oriented trajectory forecasting, practical evaluation should consider not only prediction accuracy, but also inference latency, computational resource utilization, thermal behavior, and power consumption.

The results show that deployment performance depends strongly on the availability of hardware acceleration. While the Intel i7-1260P provides higher throughput, this is achieved at the expense of considerably higher power consumption and operating temperature, whereas the Intel Celeron lacks sufficient computational capability for practical graph-based trajectory prediction. In comparison, the NVIDIA Jetson Xavier NX provides the most balanced deployment profile by combining moderate latency with stable thermal behavior, efficient power consumption, and heterogeneous GPU acceleration. These observations demonstrate that deployment-oriented evaluation jointly considers latency, computational efficiency, thermal characteristics, and energy consumption, thereby justifying the inclusion of this cross-platform deployment analysis.

### 4.7. Explainable AI-Driven Validation for Trajectory Prediction

In contrast to previous works based on black-box models, this paper proposes the integration of a well-developed explainability framework (Section 4.7.1, Section 4.7.2, Section 4.7.3 and Section 4.7.4), which explains the predictions made by the model via analysis of temporal sensitivity, neighbors’ interactions, spatial distances’ perturbations, and gradient-based feature importance.

#### 4.7.1. Temporal Sensitivity Analysis

**Figure 6:**
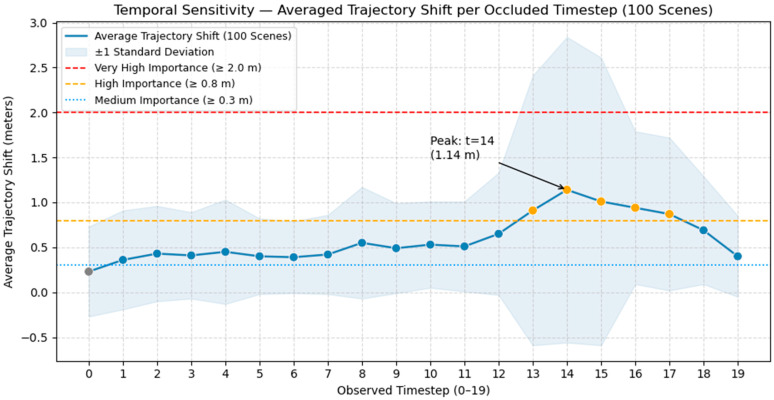
Average Temporal Sensitivity Analysis over 100 validation scenes. Blue circles represent the average trajectory shift at each observed timestep, while orange circles highlight timesteps identified as high-importance. The light blue shaded region denotes ±1 standard deviation, and the dashed horizontal lines indicate the predefined importance thresholds.

In order to evaluate whether the temporal importance observed in the original single-scene analysis represents a consistent model behavior rather than scene-specific variation, a temporal occlusion study was performed on 100 representative validation scenes from the Argoverse 1 dataset. For each scene, a reference trajectory was first predicted using the complete observation sequence. Subsequently, each observed timestep was individually occluded, and the resulting trajectory shift between the reference prediction and the occluded prediction was computed as a measure of temporal importance. This procedure was repeated independently for all 100 scenes, and the average trajectory shift together with the corresponding standard deviation was calculated for each timestep to obtain a dataset-level temporal sensitivity profile. The resulting curve is shown in Figure 6, where the *x*-axis represents the observed timesteps, the *y*-axis denotes the mean trajectory shift, and the shaded region corresponds to ±1 standard deviation across all evaluated scenes. The corresponding mean trajectory shift, standard deviation, and normalized temporal importance for each timestep are summarized in Table 6.

As illustrated in Figure 6, the averaged temporal sensitivity profile exhibits a smooth and consistent increase in importance toward the most recent observations, with the maximum average trajectory shift occurring at timestep 14 (1.14 m). The larger standard deviation observed between timesteps 13 and 18 further indicates that recent observations consistently contribute most to trajectory prediction, although the magnitude of their influence varies depending on the traffic context. Overall, these results demonstrate that the proposed framework learns a robust recency-aware temporal representation rather than relying on isolated scene-specific patterns. It should be noted, however, that the exact peak timestep and sensitivity magnitude reported here are specific to the Argoverse 1 dataset and should not be interpreted as universally applicable characteristics of vehicle behavior.

#### 4.7.2. Neighbor Interaction Sensitivity Analysis

To assess the impact of the surrounding vehicles on trajectory prediction, a neighbor occlusion analysis was conducted. For a given scene, a reference trajectory is first predicted based on the entire input. Then, for each scene, each of the surrounding vehicles is occluded individually, i.e., by zeroing the feature representation of that particular vehicle, while keeping the feature representation of the ego vehicle and all the other neighboring vehicles the same. For a given scene, a reference trajectory prediction is first obtained from the model. Following this, a neighboring vehicle is occluded from the scene, after which the model is run again to obtain a new trajectory prediction. The difference between the original and the occluded model predictions is computed as a trajectory shift, which corresponds to the influence of that particular neighbor.

**Figure 7:**
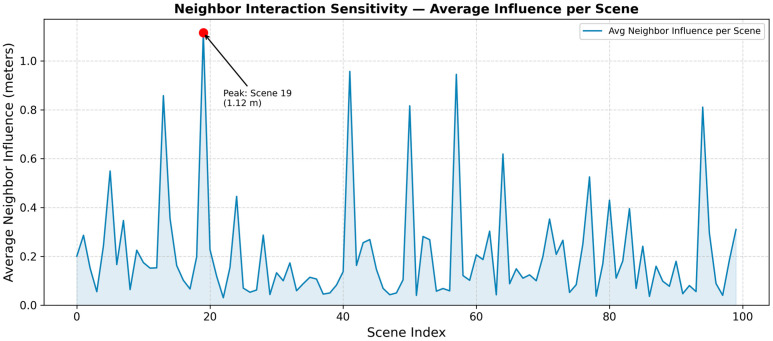
The average neighbor influence across multiple scenes.

Here, each scene gives a set of influence scores, where the number of scores is equal to the number of surrounding neighbor vehicles. These scores are combined by taking an average, which gives a combined scene-level interaction strength. This process is repeated for 100 validation scenes, providing a distribution of neighbor influence. Figure 7 illustrates the average neighbor influence over multiple scenes. As can be observed, the results show that most scenes have low interaction sensitivity, implying that the model can focus on the motion history of the ego vehicle in these scenes. However, other scenes have high peaks in the results, implying high interaction between the ego vehicle and other surrounding agents.

To give a quantitative overview of the neighbor interaction effects, the overall influence score statistics for the different validation scenes are given in the form of a statistical summary in Table 7. The results reveal that the mean neighbor influence is 0.25, indicating that the overall effect of neighboring vehicles on the prediction of the trajectory is moderate on average. Furthermore, the standard deviation of the overall neighbor influence is 0.37, indicating that the effect of neighbor interaction is highly scenario-dependent. Moreover, the maximum neighbor influence of 2.89 indicates that the model also encounters scenarios where the neighbor interaction is high, typically in complex scenarios where the traffic involves actions such as merging or lane-changing. However, the minimum neighbor influence of 0.03 indicates that the overall effect of neighbor interaction is low in most scenarios, where the model relies on the motion history of the ego vehicle to make the prediction of the trajectory.

#### 4.7.3. Spatial Distance-Based Sensitivity Analysis

**Figure 8:**
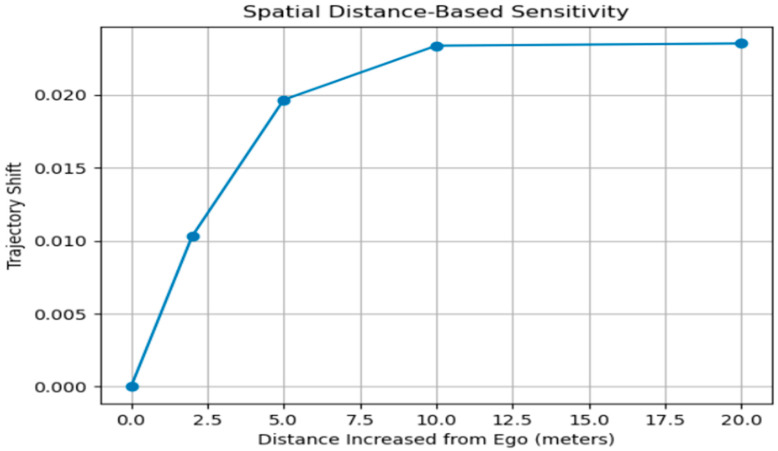
Spatial distance-based sensitivity analysis.

In order to investigate the geographical range of neighbor impacts on ego trajectory prediction, a distance-based perturbation analysis was carried out using increasing distances of 0, 2, 5, 10, and 20 m between the neighboring car and the ego car in their connection direction, followed by a calculation of the displacement using average Euclidean distance to the reference prediction. It was shown in Figure 8 and Table 8 that the displacement of the trajectory is in the form of a saturation logarithmic curve, not a straight line or unbounded increase. A rapid increase in sensitivity is observed within the 0–5 m range, where the trajectory shift rises from 0.00 m to 0.01 m, indicating that the model is highly sensitive to nearby vehicles.

From beyond 5 m, the marginal effect gradually diminishes, decreasing to +0.0037 m from 5 to 10 m and then further to an insignificant +0.0001 m from 10 to 20 m, corresponding to a decline of about 99 percent relative to the first increment, indicating that interaction saturation occurs within 10 m of the ego vehicle. The observed saturation effect is reasonable in light of the distance-based attention function in the Actor-to-Actor component, which automatically assigns lower attention scores to vehicles that are farther away, reducing their contribution to the ego agent’s feature representation. The minimal variation of 0.0001 m across the two distance ranges provides evidence that the proposed model effectively prevents any unreasonable impact of distant vehicles.

#### 4.7.4. Gradient-Based Timestep Sensitivity Analysis

**Figure 9:**
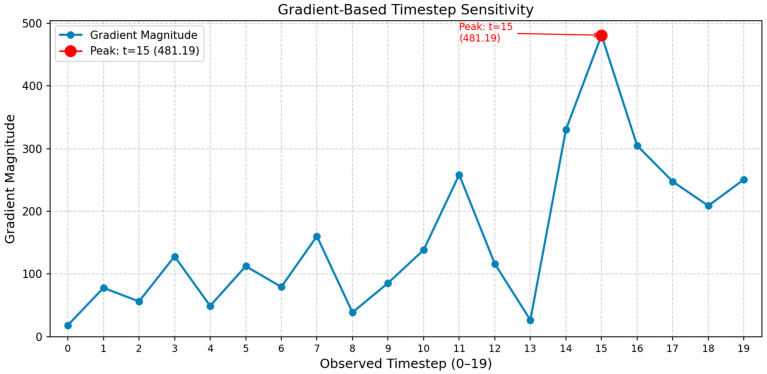
The gradient-based timestep sensitivity analysis.

In order to gain deeper insight into the mechanism through which the model incorporates temporal and feature-level information from previous motion, a sensitivity analysis based on gradients is conducted. Different from the perturbation analysis method, this technique does not manipulate the input data directly; rather, it derives the gradient of the generated motion path with regard to the input motion feature data. In this test, the gradient tracking for motion features is enabled, and a scalar objective function is constructed using the generated motion path associated with the maximum probability mode. The gradient values are derived through the backpropagation process and are summed up with respect to the absolute values to obtain the sensitivity values at each timestep. As shown in Figure 9, the gradient values differ greatly at different timesteps, revealing that the temporal sensitivity of the output varies greatly, with smaller values at earlier timesteps and larger ones at later timesteps.

The gradient analysis displays a sharp peak at timestep 15, which possesses the highest value of the gradient with a magnitude of 481.19. Thus, the current model demonstrates the highest sensitivity to this specific point in the input sequence. Other timesteps such as 14, 16, and 11 also display comparatively high values of the gradient, which indicates the presence of dependency on recent motion characteristics rather than equal attention to all timesteps. For statistical interpretation of these results, Z-scores are used to normalize the obtained values, as shown in Table 9 below. Z-score represents the difference between the value and the mean in terms of standard deviations, which allows establishing the importance of each point relative to others. Timestep 15 holds the highest value of the Z-score (+1.89), which confirms the presence of a significant positive impact on the model output.

In order to get a statistical explanation, Z-score normalization has been performed on the gradient values as presented in Table 9. The Z-score at each timestep has been calculated using the formula,

$$Z-score=\frac{\left(X\;-\;\mu \right)}{\sigma }$$

Here x stands for the gradient value at the respective time step, μ denotes the mean value for all gradients, while σ signifies the standard deviation of the entire data set. The above calculation helps in determining the extent to which a particular timestep varies from the mean sensitivity in terms of standard units. Time Step 15 possesses a Z-value of +1.89, suggesting that it is highly significant since it is positively distant from the mean value.

**Figure 10:**
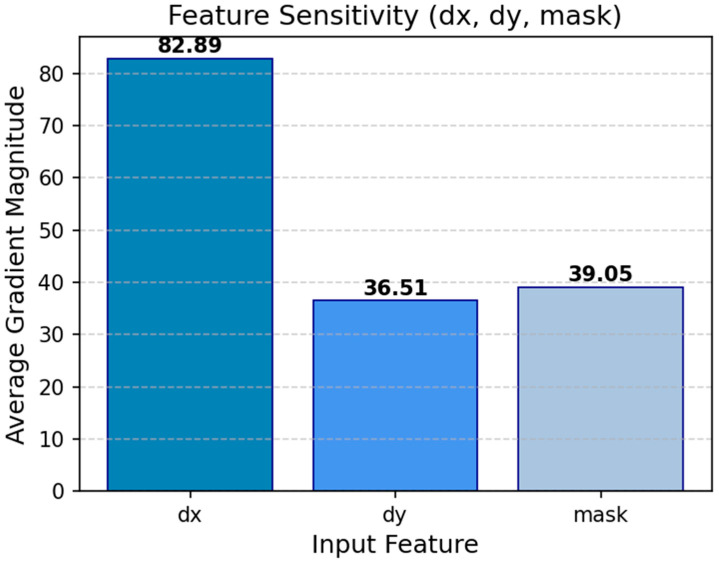
Feature-wise gradient sensitivity analysis.

Figure 10 presents the feature-wise gradient sensitivity analysis, illustrating the relative importance of each input motion feature horizontal displacement (dx), vertical displacement (dy), and the validity mask—in influencing the model’s trajectory prediction. The lateral position change feature dx signifies the difference in the side-to-side position of the vehicle between frames and serves as the most relevant behavior-wise signal for lane changes. The longitudinal position change feature dy reflects progress along the lane change direction. The feature mask is a boolean value indicating whether the motion values from a given timestep can be considered valid or are merely added as padding due to lack of data at the respective timestep. As demonstrated in Figure 8, dx has the highest magnitude average gradient of 82.89, significantly surpassing dy (36.51) and mask (39.05).

The overall statistical summary of the gradient sensitivity analysis is presented in Table 10, including the mean, maximum, and minimum gradient values, as well as the most influential timestep.

### 4.8. Trajectory Mode Selection Analysis

In order to understand more about how the trajectory prediction model is able to determine the most likely trajectory, a mode selection experiment was carried out. The trajectory forecasting model is able to predict various possible trajectories for a given vehicle. These trajectories represent various possible motion patterns. These trajectories are referred to as prediction modes. Each prediction mode is assigned a confidence score that represents how likely a particular motion is. The best mode is referred to as the trajectory with the best confidence score, and alternative prediction is referred to as the trajectory with the second-best confidence score.

**Figure 11:**
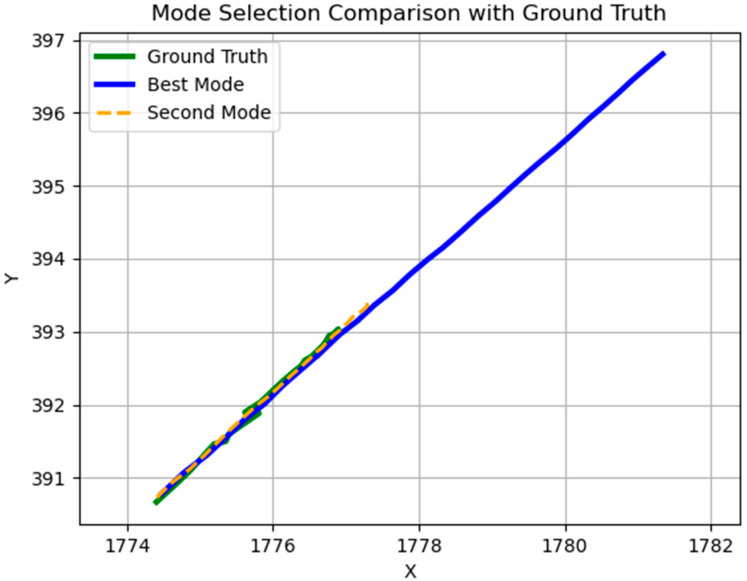
Visualization of multimodal trajectory prediction.

Figure 11 is a representation that compares the best mode and second mode predicted trajectories in relation to the actual ground truth trajectory for the movement of the vehicle. From Figure 9, it is also clear that the green line denotes the ground truth, and the blue line and dashed orange line denote the best predicted trajectory and second predicted trajectory, respectively.

Since the trajectories predicted by the model overlap considerably with the ground truth trajectory, we can conclude that the model predicts accurately with minimum uncertainty. As seen from the figure, both the predicted trajectories follow the true trajectory to an extent where the first mode is almost perfectly aligned while the second mode shows only minor deviation. The close proximity of the predicted modes suggests that there is very little model uncertainty in predicting the future movement of the vehicle.

### 4.9. Noise Sensitivity Analysis

In order to test the robustness of the trajectory prediction model, a noise sensitivity analysis is carried out. In this experiment, different levels of Gaussian noise are added to the input motion features, and the resulting change in the predicted trajectory is measured. The level of noise is determined by the value of the standard deviation (σ), and the change in the trajectory is measured by calculating the average Euclidean distance between the original predicted trajectory and the trajectory obtained from noisy inputs.

**Figure 12:**
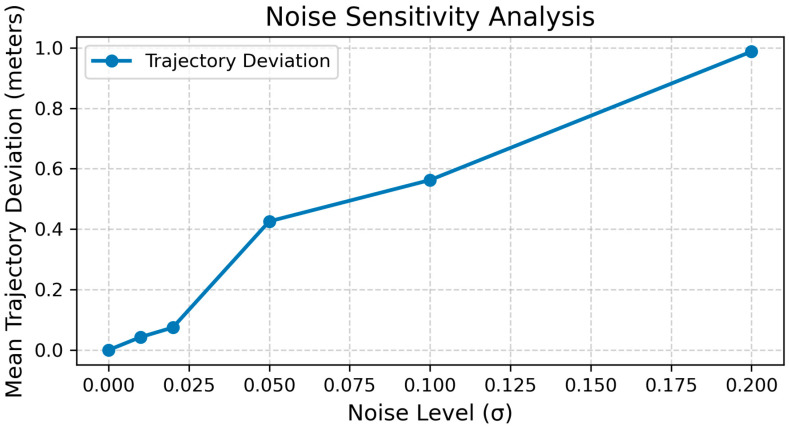
Noise sensitivity analysis.

From Figure 12, it can be observed that there is a clear relationship between the noise level and the deviation in the predicted trajectory. For low levels of noise, when σ ≤ 0.02, the deviation in the trajectory is extremely low, which means that the model is highly stable even in the presence of low levels of perturbations in the input. It can also be observed that even when the noise level increases slightly to σ = 0.05, there is only a low increase in the deviation. However, as the noise level increases beyond this range, a significant increase in trajectory deviation is observed, indicating that the model is becoming more sensitive to disturbances in the input motion features. Yet, this increase in deviation is not extreme, and a controlled level of deviation is maintained, even at higher levels of noise (σ = 0.2). This, in essence, indicates that the model is handling noise effectively without any unstable results.

The relationship between input noise and trajectory deviation is presented in Table 11. It can be observed that when the level of noise is low, robustness remains high. There is a notable transition in the model when σ = 0.05, beyond which sensitivity improves. However, it should be noted that the rate of increase in deviation remains low, which indicates stability in the model. It can be observed that the model has achieved a good balance between robustness and sensitivity.

### 4.10. Mode Confidence Distribution Analysis

**Figure 13:**
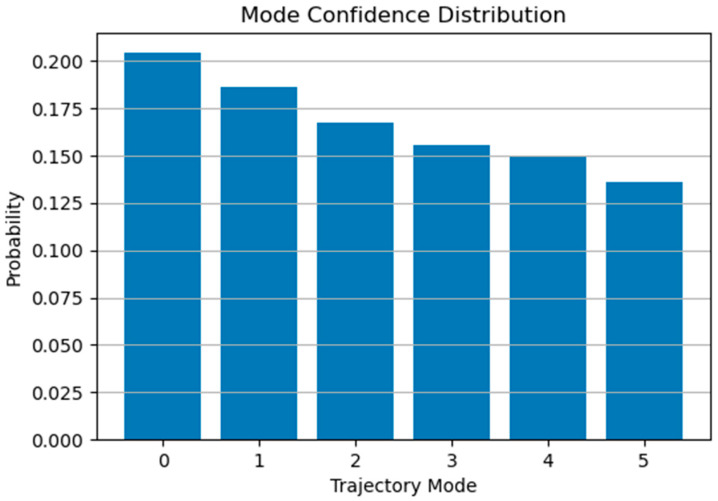
Mode confidence distribution analysis.

In order to comprehend how the model rates different possible future paths, a mode confidence analysis was carried out. In the trajectory prediction model, multiple possible paths (modes) are created for each vehicle, and a probability score is provided for each mode. These probability scores indicate the model’s confidence in each of the possible paths that are predicted. Figure 13 illustrates the probability scores of the different modes of the predicted paths. In the figure, the horizontal axis refers to the different modes of the paths, and the vertical axis refers to the probability.

From Figure 13, it is observed that Mode 0 has the maximum probability, which means that the model has taken this particular trajectory as the most likely future trajectory. The confidence values of the model are decreasing gradually for the other modes, with Mode 5 having the least probability value. This shows that the model has ranked the possible modes according to their probability values, with the most probable motion pattern having the most importance. The difference in the values of confidence between the two modes can give some additional information about the model’s level of uncertainty. In this particular example, the values of the probabilities are close to each other, and this indicates that although one of the modes is preferred, the model still considers other possible trajectories as feasible. This is due to the level of uncertainty involved in trajectory prediction.

As a result, the overall performance of the mode confidence distribution is such that it effectively prioritizes the most probable trajectory while at the same time providing multiple alternative predictions. This is important in real-world driving situations where the behavior of the vehicle may change depending on the environment. Table 12 represents the probability distribution of the predicted trajectory modes. It can be seen that the maximum probability is associated with Mode 0, which is 0.20. This represents the highest probability of the predicted trajectory. The probabilities decrease gradually with the increase in the mode numbers. This represents the ranking of the trajectory possibilities in the order of their probabilities. However, the probabilities are not drastically different from each other, representing the uncertainty of the model in the trajectory possibilities.

### 4.11. Multimodal Trajectory Prediction Analysis

In order to understand how the model predicts possible future behaviors, a multimodal trajectory visualization was performed. The trajectory prediction model predicts several possible future trajectories (modes) for the ego vehicle, and each mode represents a possible motion pattern. In addition, the model predicts a confidence score for each of these trajectories. The confidence score is converted into a probability using a softmax function.

**Figure 14:**
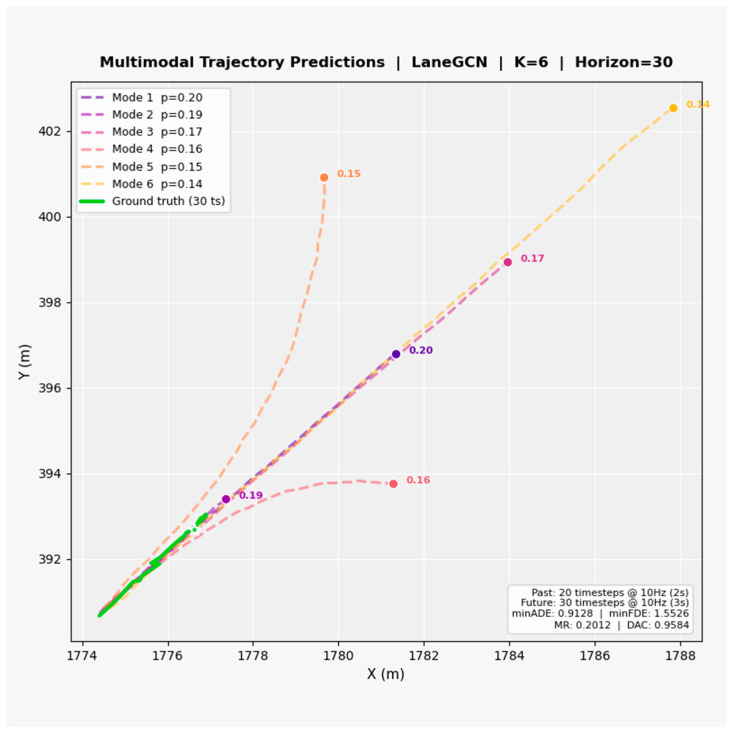
Visualization of multimodal trajectory predictions.

Figure 14 shows the predicted trajectories for all modes, as well as the ground truth. The x and y axes of the plot represent the spatial coordinates of the vehicle. The predicted trajectories are drawn with varying levels of transparency based on their probabilities. The trajectories with higher probabilities appear more prominent. In addition, the probabilities for each mode are also shown near the end of their respective trajectories.

From this figure, it can be seen that various trajectories tend to move in a similar general direction, while a few tend to deviate slightly, representing different possible future paths that the moving object might take. The path with the highest probability tends to move in a similar direction to the ground truth path, indicating that the model is able to correctly identify the most probable future path that the moving object is going to take. The various trajectories also indicate that the model is able to handle various possible paths that the moving object might take, rather than sticking to just one path. The various values associated with each path also indicate that the model is able to rank all the possible paths according to their individual probabilities. The small range associated with all the trajectories indicates that this is not a complex situation, and the moving object is more likely to take a consistent path.

### 4.12. Ablation Study

The complete ablation study results for all five model configurations are listed in Table 13, which measures the performance on four standard trajectory prediction evaluation criteria: minimum Average Displacement Error, minimum Final Displacement Error, Miss Rate, and Drivable Area Compliance. Lower values are better for the first three criteria, and higher values are better for the fourth.

The Key observations from the ablation study are

(1) Spatial Dominance of LaneGAT: The performance improvement from TCN Only with minADE = 1.53 to LaneGAT Only with minADE = 1.03 is a significant improvement of 32.6%. This shows that spatial information is more important than temporal information for accurate trajectory prediction in a structured driving environment.
(2) Fusion of TCN and LaneGAT: The performance improvement from TCN + LaneGAT with minADE = 0.97 to TCN Only with minADE = 1.53 and LaneGAT Only with minADE = 1.03 shows that our fusion method is able to effectively utilize temporal momentum for accurate spatial pathfinding.
(3) Full Model Superiority: The full model, which consists of the TCN, LaneGAT, and MHSA, performs the best in terms of minADE (0.91), minFDE (1.55), and Miss Rate (0.2012), which corresponds to a 40.5% decrease in ADE and a 63.2% decrease in Miss Rate relative to the baseline.
(4) DAC Trade-off: The combination of Multi-Head Self-Attention and TCN produces the best DAC score of 0.97, which implies the effectiveness of social attention in ensuring the satisfaction of the drivable area constraint, even if the accuracy of the coordinates is not the best.

This ablation study confirms every architectural choice in the framework. LaneGAT supplies the structural component by learning the topology of the map, while TCN offers the critical motion context with temporal modeling, and MHSA plays the role of the global social refiner, which minimizes the Miss Rate while maintaining competitive performance in DAC. These enhancements prove the efficacy of the combined model, verifying the optimal performance of the combined model for autonomous vehicle trajectory prediction.

**Figure 15:**
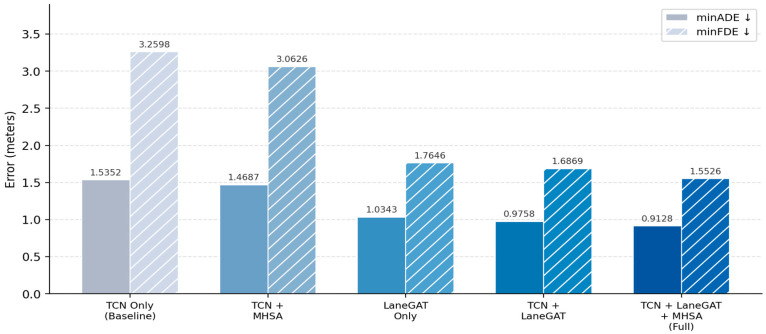
Comparison of minADE and minFDE across all model configurations. The downward arrow (↓) indicates that lower values correspond to better prediction performance. Different shades are used solely to distinguish the evaluated model configurations for improved readability.

**Figure 16:**
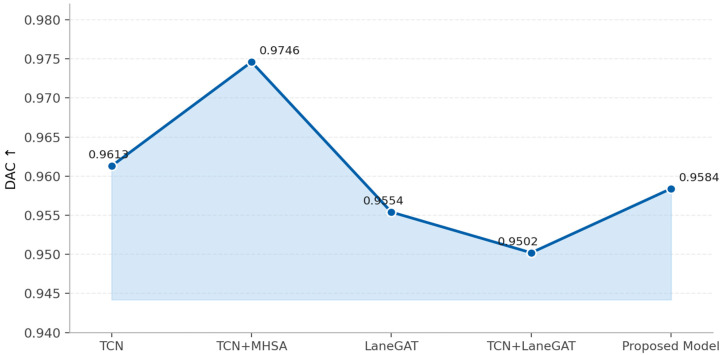
Comparison of Drivable Area Compliance (DAC) across different model configurations. The upward arrow (↑) indicates that higher DAC values correspond to better performance. The shaded area is used solely to improve the visual readability of the figure.

The following Figure 15 and Figure 16 offer the visual representations of the results from the ablation study.

### 4.13. Impact of Training Data Size on Model Performance

In order to analyze the effect of the size of training data on performance, three different ratios of the training data were analyzed through experiments, as depicted in Table 14 below: 30%, 50%, and 100%. From the analysis conducted at the 45th epoch, it is evident that the performance of the model improves with an increase in the amount of training data. In the case when only 30% of the data is considered for training, the model has high prediction errors, with ADE being equal to 1.59 m, FDE—to 3.38 m, and MR being 0.56. In the case of an increase in the percentage of the dataset up to 50%, a significant improvement in results can be achieved because ADE becomes 0.98 m and FDE equals 1.66 m. The MR becomes 0.22 at that. The best model accuracy is demonstrated with the use of the 100% dataset when ADE becomes 0.90 m, FDE equals 1.50 m, and MR becomes 0.194. However, the DAC stays high in all cases.

## 5. Discussion

The experimental results indicate that trajectory forecasting performance in structured urban environments is strongly influenced by lane topology awareness and localized interaction reasoning rather than temporal modeling alone. Although transformer-based approaches such as ADAPT and SIMPL achieve lower displacement errors on the Argoverse benchmark, these methods rely on computationally intensive global attention operations evaluated primarily on high-performance server-grade GPUs. In contrast, the present study focuses on a different objective: investigating whether interpretable graph-based trajectory forecasting can simultaneously achieve reliable forecasting performance and model interpretability, while remaining feasible for embedded autonomous driving deployment. The obtained performance of 0.90 m minADE and 1.50 m minFDE, therefore, represents a deliberate operating point that balances forecasting accuracy with deployment feasibility rather than an attempt to maximize benchmark accuracy alone. The ablation experiments further highlight the relative contribution of spatial and temporal reasoning components. The substantial improvement observed when introducing lane-aware graph reasoning over the TCN-Only baseline confirms that road topology acts as a dominant structural prior for vehicle motion prediction in lane-constrained traffic environments. This observation is consistent with real-world driving behavior, where future vehicle motion is typically restricted by lane geometry and road connectivity. Temporal modeling through TCN and MHSA contributes additional improvements by emphasizing behavior-critical motion patterns and suppressing temporally redundant observations. The reduction in minADE from 1.53 m in the temporal-only baseline to 0.90 m in the complete framework suggests that temporal attention is particularly beneficial during maneuver preparation phases such as lane adjustments and local trajectory corrections.

While the proposed framework employs considerably fewer trainable parameters (1.96 M) than the transformer-based baselines compared in Table 2 (2.65 M–13.1 M), the experimental results indicate that the reduced model complexity does not proportionally degrade forecasting performance for structured urban driving scenarios. In particular, the ablation study presented in Table 13 shows that the Drivable Area Compliance (DAC) remains consistently high (0.95–0.96) across all model configurations, suggesting that the lane-aware graph representation provides a strong structural inductive bias that compensates for the reduced parameter budget. Nevertheless, this compact architecture introduces an inherent trade-off. Compared with larger transformer-based models, the proposed framework exhibits higher displacement errors in scenarios involving long-range dependencies and dense multi-agent interactions, where global attention mechanisms provide greater representational flexibility. Consequently, the proposed design intentionally prioritizes deployment efficiency and model interpretability while maintaining reliable forecasting performance for resource-constrained embedded autonomous driving systems.

The additional deployment comparison with recent trajectory prediction models further strengthens the practical positioning of the proposed framework. While transformer-based approaches such as HiVT and SIMPL achieve lower displacement errors on the Argoverse benchmark, the Jetson Xavier NX evaluation demonstrates that these accuracy gains are accompanied by increased computational cost during embedded deployment. Compared with HiVT and SIMPL, the proposed framework achieves the lowest inference latency, highest effective throughput, and lower CPU utilization while maintaining stable thermal and power characteristics. These results demonstrate that the reduction in model complexity translates directly into measurable improvements in embedded deployment efficiency under identical hardware conditions. Consequently, the proposed framework occupies a practical operating point in the accuracy–deployment trade-off space, where moderate reductions in forecasting accuracy enable substantially improved deployment characteristics on embedded autonomous driving platforms.

The explainability analyses provide additional insight into the temporal and interaction dynamics learned by the model. Both temporal occlusion and gradient-based attribution consistently identify timesteps 14–16 as the most influential region within the observation horizon. This suggests that maneuver-related behavioral cues become increasingly distinguishable approximately 1.5 s before the prediction interval, which aligns with the gradual nature of human driving decisions during lane adjustment and speed adaptation. Furthermore, the substantially higher attribution associated with lateral displacement compared to longitudinal motion indicates that lateral trajectory variation contains stronger predictive information for future maneuver estimation in structured road environments.

The additional multi-scene temporal sensitivity analysis further strengthens the interpretability findings. While single-scene explanations can contain localized fluctuations arising from specific traffic configurations, averaging the temporal occlusion responses across 100 validation scenes reveals a stable temporal importance pattern in which recent observations consistently exert the greatest influence on future trajectory prediction. This result indicates that the proposed temporal attention mechanism captures persistent behavioral characteristics rather than isolated frame-specific artifacts. Nevertheless, improving temporal explanation consistency through attention regularization and temporally smooth attribution mechanisms remains an important direction for future research.

The interaction sensitivity experiments reveal that neighboring vehicle influence is highly dependent on traffic complexity and local maneuver density. In many normal driving scenarios, neighboring agents contribute only limited trajectory perturbation. However, during merging and lane-changing situations, interaction sensitivity increases significantly, with trajectory influence reaching up to 2.89 m. Similarly, the spatial perturbation analysis indicates that interaction relevance decreases beyond approximately 10 m, suggesting that the model implicitly learns localized interaction priors instead of relying on unrestricted global correlations. This behavior is particularly important for autonomous driving systems, where excessive dependence on distant agents may introduce unstable or physically inconsistent trajectory forecasts. From a deployment perspective, the experimental findings highlight the practical challenges associated with executing graph-based trajectory forecasting architecture on embedded edge hardware. Unlike conventional forecasting studies that primarily optimize displacement accuracy on high-end GPUs, the present work additionally investigates thermal stability, resource utilization, latency, and power efficiency under realistic deployment constraints. The Jetson Xavier NX evaluation demonstrates that stable trajectory inference can be achieved within moderate power and thermal limits despite the dynamic graph operations inherent in LaneGCN-based architectures. The deployment-aware optimizations, including mixed-precision inference, graph simplification, adaptive lane selection, and temporal downsampling, contribute toward reducing graph computation overhead while preserving behavior-relevant forecasting information. Future improvements in embedded trajectory forecasting may combine local model optimization with intelligent computation-offloading strategies that dynamically allocate latency-critical tasks across heterogeneous edge-computing resources, thereby further improving deployment efficiency under varying computational workloads [38]. These observations highlight that deployment-oriented optimization should be considered alongside forecasting accuracy when evaluating practical autonomous driving systems.

The multimodal forecasting and robustness experiments further suggest that the framework maintains stable prediction behavior under moderate perturbation conditions. The generated trajectories remain physically plausible under Gaussian noise disturbances, indicating that the learned motion representations are not overly sensitive to small observation inconsistencies. This robustness is particularly relevant in practical autonomous driving systems where localization uncertainty, sensor noise, and environmental disturbances are unavoidable.

Nevertheless, several limitations remain. The forecasting accuracy still lags behind large-scale transformer-based architectures in highly interactive or long-range prediction scenarios, indicating that global context aggregation remains beneficial for complex traffic understanding. In addition, the current deployment strategy does not incorporate advanced graph acceleration frameworks or hardware-specific graph compilation techniques. Future work can therefore investigate lightweight graph transformers, sparse interaction mechanisms, uncertainty-aware trajectory forecasting, and dedicated embedded graph inference optimization for large-scale deployment scenarios. Recent linear-complexity State-Space Models (SSMs) have demonstrated promising efficiency characteristics for long-sequence temporal modeling and can provide an alternative to attention-based forecasting architectures for resource-constrained autonomous driving systems. However, the present work focuses primarily on investigating interpretable graph-based trajectory forecasting with explicit temporal importance modeling and embedded deployment feasibility. Integration of lightweight SSM-based temporal encoders with lane-aware interaction reasoning remains an important future research direction.

The present study focuses primarily on perturbation-based and gradient-based interpretability analyses due to their computational feasibility for graph-based forecasting architectures. More computationally intensive causal and game-theoretic explainability approaches, including Shapley-value-based attribution and counterfactual trajectory reasoning, remain important future directions for validating behavioral causality and interaction significance in autonomous driving trajectory prediction.

## 6. Conclusions and Future Works

This paper presented an interpretable and edge-deployable spatio-temporal trajectory prediction framework for autonomous driving, focusing on the balance between forecasting accuracy, deployment feasibility, and explainability under embedded resource constraints. Experimental evaluation on the Argoverse 1 benchmark demonstrated practical forecasting performance with a minADE of 0.90 m, minFDE of 1.50 m, Miss Rate of 0.19, and DAC of 0.95. In addition to trajectory prediction accuracy, the proposed framework was evaluated on the NVIDIA Jetson Xavier platform, achieving practical embedded inference capability with 7.95 samples/s at an average power consumption of 12.86 W. Furthermore, additional deployment experiments performed using transformer-based approaches such as HiVT and SIMPL under identical NVIDIA Jetson Xavier NX conditions demonstrated that the proposed framework achieves a more favorable balance between forecasting performance and embedded deployment efficiency than recent transformer-based trajectory prediction models. The explainability analyses further demonstrated that the model captures behaviorally meaningful temporal and spatial patterns, with temporal sensitivity, lateral motion behavior, and localized neighboring interactions contributing strongly to forecasting decisions.

Despite these contributions, the work has several limitations that should be acknowledged. The model is evaluated exclusively on the Argoverse 1 dataset covering Pittsburgh and Miami; its generalization to different driving environments, road types, and geographic regions remains untested. The current framework is evaluated using a fixed 3 s prediction horizon, which may limit long-range forecasting capability in high-speed highway scenarios. The dynamic graph operations in LaneGCN prevent the application of standard quantization techniques such as TensorRT and INT8, meaning that further hardware-level acceleration through conventional static quantization pipelines remains challenging without architectural modifications. The current multimodal Prediction Head generates a fixed number of trajectory hypotheses irrespective of scene ambiguity, which may not fully capture uncertainty variation across different traffic conditions. Additionally, the current framework operates in open-loop prediction mode and has not been integrated or evaluated within a closed-loop planning and control pipeline, which limits conclusions about its practical impact on driving safety metrics. Although the current latency remains above the stringent requirements of tightly coupled Level-4 autonomous driving control loops, the proposed framework establishes a practical baseline for embedded trajectory forecasting and motivates further hardware-aware optimization.

It is also important to note that the interpretability findings, including the peak average temporal sensitivity at timestep 14 observed in the averaged analysis over 100 validation scenes, and the approximately 10 m interaction saturation distance, were derived exclusively from the Argoverse 1 dataset (Pittsburgh and Miami) and should not be interpreted as universally applicable values. The broader qualitative observations, namely that recent motion history and lateral displacement contribute more strongly to trajectory prediction than earlier observations, and that neighboring vehicle influence decreases with increasing spatial separation, are consistent with established vehicle motion characteristics and previous trajectory forecasting studies. Nevertheless, the quantitative values reported in this work remain dataset and scenario-dependent, and their generality across different traffic environments has not been experimentally validated. Cross-dataset evaluation using datasets such as nuScenes, Waymo Open Motion, and Indian unstructured-traffic datasets therefore represents an important direction for future work. Future work shall focus on improving both forecasting scalability and embedded deployment efficiency. Future deployment optimization shall additionally investigate lightweight graph representations, static graph compilation strategies, and hardware-aware graph acceleration techniques to improve inference efficiency on embedded autonomous driving platforms. Structured model pruning and knowledge distillation will also be investigated to further reduce inference latency on embedded autonomous driving platforms. Integrating lightweight state-space temporal models and adaptive sparse interaction mechanisms may further improve scalability under resource-constrained conditions. Furthermore, extending the framework toward closed-loop autonomous driving evaluation with planning-aware safety metrics, collision avoidance analysis, and uncertainty-aware trajectory reasoning remains an important future research direction. Future research will investigate temporally consistent attention regularization and explanation-aware training strategies to further improve the robustness and stability of temporal interpretability under individual frame perturbations. Finally, incorporating real-time confidence estimation and causal explainability mechanisms may improve transparency and reliability in safety-critical autonomous driving applications.

## Figures and Tables

**Table 1:** Quantitative performance of the proposed model in the Argoverse 1 evaluation dataset.

Metric (K = 6)	Value
minADE (m)	0.90
minFDE (m)	1.50
Miss Rate (MR)	0.19
Drivable Area Compliance (DAC)	0.95

**Table 2:** Comparison of transformer-based methods with the proposed method on the Argoverse 1 dataset.

Method	Interaction Modeling Scope	Edge Evaluated	MinADE (m)	MinFDE (m)	MR	Model Parameters
Scene Transformer [14]	Global spatio-temporal attention	**No**	0.80	1.23	0.13	13.1 M
MmTransformer [39]	Global multi-agent attention	**No**	0.71	1.15	0.21	4.4 M
ADAPT [40]	Scene-centric adaptive attention	**No**	0.67	0.95	0.08	3.1 M
SIMPL [27]	Symmetric global interaction fusion	**No**	0.66	0.95	0.08	2.65 M
HiVT [12]	Hierarchical local-global interaction	**No**	0.66	0.96	0.19	6.1 M
**Proposed Approach** (**Ours**)	Local temporal attention + lane-aware graph interaction	**Yes**	0.90	1.50	0.19	1.96 M

**Table 3:** Edge deployment performance evaluation on NVIDIA Jetson Xavier NX platform.

Metric	Measured Value	Unit	Deployment Interpretation
**INFERENCE PERFORMANCE**			
Average Latency	**125.74**	ms/batch	The model achieves low inference latency, supporting responsive trajectory prediction for edge deployment.
Throughput	**1.99**	batches/s	Stable batch inference under embedded execution constraints.
Effective FPS	**7.95**	samples/s	The achieved frame processing rate indicates practical feasibility for near real-time autonomous driving inference.
**RESOURCE UTILIZATION**			
CPU Usage	**57.34**	%	Moderate CPU utilization indicates balanced host-side computational demand without excessive processor load.
RAM Usage	**71.43**	%	Moderate memory utilization within operational limits.
GPU Usage	**55.88**	%	Moderate GPU utilization implies efficient usage of the accelerator with available computing resources.
**THERMAL & POWER**			
Average Temperature	**58.92**	°C	Stable thermal behavior without throttling.
Average Power	**12.86**	W	Low power consumption indicates energy-efficient operation.

**Table 4:** Comparative edge deployment analysis with recent trajectory prediction models.

Metric	Proposed Model	HiVT [12]	SIMPL [27]
Average Latency (ms/sample)	125.73	151.09	187.60
Throughput (batches/s)	1.99	1.65	1.33
Effective FPS (samples/s)	7.95	6.62	5.33
Avg CPU Usage (%)	57.34	73.28	94.18
Avg GPU Usage (%)	55.88	65.79	60.46
Avg RAM Usage (%)	71.43	82.41	74.94
Avg Temperature (°C)	58.92	69.46	55.93
Avg Power Draw (W)	12.86	12.50	11.81
minADE (m)	0.90	0.66	0.66

**Table 5:** Cross-platform deployment evaluation of the proposed trajectory prediction framework.

Metric	Jetson Xavier NX	i7-1260P	Celeron
Latency per Sample (ms/sample)	125.73	195.18	595.72
Throughput (batches/s)	1.99	2.60	1.68
Effective FPS (samples/s)	7.95	5.20	1.68
Avg CPU Usage (%)	57.34	84.08	99.91
Avg GPU Usage (%)	55.88	0.00	0.00
Avg RAM Usage (%)	71.43	47.94	65.57
Avg Temperature (°C)	58.92	70.44	69.48
Peak Temperature (°C)	64.20	100	72.10
Avg Power Draw (W)	12.86	25.55	7.85

**Table 6:** Average temporal sensitivity analysis across 100 validation scenes.

Masked Timestep	Mean Trajectory Shift (m)	Standard Deviation (m)	Importance (%)
0	0.23	0.50	20.18
1	0.36	0.55	31.58
2	0.43	0.53	37.72
3	0.41	0.48	35.96
4	0.45	0.58	39.47
5	0.40	0.42	35.09
6	0.39	0.40	34.21
7	0.42	0.44	36.84
8	0.55	0.62	48.25
9	0.49	0.50	42.98
10	0.53	0.48	46.49
11	0.51	0.50	44.74
12	0.65	0.68	57.02
13	0.91	1.50	79.82
**14**	**1.14**	**1.70**	**100.00**
15	1.01	1.60	88.60
16	0.94	0.85	82.46
17	0.87	0.85	76.32
18	0.69	0.60	60.53
19	0.40	0.45	35.09

Bold value indicated the timestep exhibiting the maximum average trajectory shift and the highest temporal importance across the validation scenes.

**Table 7:** Statistical summary of neighbor interaction influence across 100 validation scenes.

Metric	Value (m)
Mean Influence	0.25
Standard Deviation	0.37
Maximum Influence	2.89
Minimum Influence	0.03

**Table 8:** Effect of spatial distance on trajectory shift.

Distance Increase from Ego (m)	Trajectory Shift (m)	Incremental Sensitivity (m)	Interaction Zone
**0**	0.0000	--	Baseline
**2**	0.0103	0.0103	High local sensitivity
**5**	0.0197	0.0094	Reduced sensitivity
**10**	0.0234	0.0037	Saturation transition
**20**	0.0235	0.0001	Saturated response

**Table 9:** Major influential timesteps based on gradient sensitivity.

Rank	Timestep (s)	Gradient Value	Z-Score
1	15	481.19	+1.89
2	14	330.62	+0.07
3	16	304.93	−0.25
4	11	258.52	−0.81
5	19	250.83	−0.90

**Table 10:** Statistical summary of gradient sensitivity.

Metric	Value
Mean Gradient	158.45
Maximum Gradient	481.19
Minimum Gradient	18.19
Most Influential Timestep	15

**Table 11:** Noise level vs. trajectory deviation.

Noise Level (σ)	Trajectory Change (m)
0.00	0.00
0.01	0.07
0.02	0.07
0.05	0.11
0.10	0.69
0.20	0.92

**Table 12:** Mode probability distribution for multimodal trajectory prediction.

Mode	Prediction Probability
0	0.20
1	0.18
2	0.16
3	0.15
4	0.14
5	0.13

**Table 13:** Ablation study results for different model configurations based on ADE, FDE, MR, and DAC metrics.

Configuration	MinADE (m)	MinFDE (m)	MR	DAC
TCN Only	1.53	3.25	0.54	0.96
TCN + MHSA	1.46	3.06	0.52	0.96
LaneGAT Only	1.03	1.76	0.23	0.95
TCN + LaneGAT	0.97	1.68	0.23	0.96
TCN + LaneGAT + MHSA (proposed model)	0.90	1.50	0.19	0.96

**Table 14:** Performance analysis of trajectory prediction with varying training dataset sizes.

Training Dataset	ADE (m)	FDE (m)	MR	DAC
30% of total training dataset	1.59	3.38	0.56	0.97
50% of total training dataset	0.98	1.66	0.22	0.95
Total training dataset—208,272	0.90	1.50	0.19	0.96

## Data Availability

The dataset used in this manuscript is the publicly available Argoverse 1 dataset. The corresponding dataset link is provided below: https://www.argoverse.org/av1.html#download-link (accessed on 3 July 2026).
